# Direct *in situ* spectroscopic evidence of the crucial role played by surface oxygen vacancies in the O_2_-sensing mechanism of SnO_2_†

**DOI:** 10.1039/d2sc01738e

**Published:** 2022-05-05

**Authors:** Stefan Kucharski, Pilar Ferrer, Federica Venturini, Georg Held, Alex S. Walton, Conor Byrne, James A. Covington, Sai Kiran Ayyala, Andrew M. Beale, Chris Blackman

**Affiliations:** Department of Chemistry, University College London 20 Gower St WC1H 0AJ London UK c.blackman@ucl.ac.uk; Research Complex at Harwell, Rutherford Appleton Laboratory OX11 0FA Harwell Didcot UK; Diamond Light Source, Rutherford Appleton Laboratory OX11 0FA Harwell Didcot UK; Department of Chemistry, University of Manchester M13 9PL Manchester UK; Photon Science Institute, University of Manchester M13 9PL Manchester UK; School of Engineering, University of Warwick CV4 7AL Coventry UK

## Abstract

Conductometric gas sensors (CGS) provide a reproducible gas response at a low cost but their operation mechanisms are still not fully understood. In this paper, we elucidate the nature of interactions between SnO_2_, a common gas-sensitive material, and O_2_, a ubiquitous gas central to the detection mechanisms of CGS. Using synchrotron radiation, we investigated a working SnO_2_ sensor under operando conditions *via* near-ambient pressure (NAP) XPS with simultaneous resistance measurements, and created a depth profile of the variable near-surface stoichiometry of SnO_2−*x*_ as a function of O_2_ pressure. Our results reveal a correlation between the dynamically changing surface oxygen vacancies and the resistance response in SnO_2_-based CGS. While oxygen adsorbates were observed in this study we conclude that these are an intermediary in oxygen transport between the gas phase and the lattice, and that surface oxygen vacancies, not the observed oxygen adsorbates, are central to response generation in SnO_2_-based gas sensors.

## Abstract 2

NAP-XPS characterisation of SnO_2_ under operando conditions shows that resistance change, band bending and surface O-vacancy concentration are correlated with ambient O_2_ concentration, challenging current preconceptions of gas sensor function.

## Graphical abstract

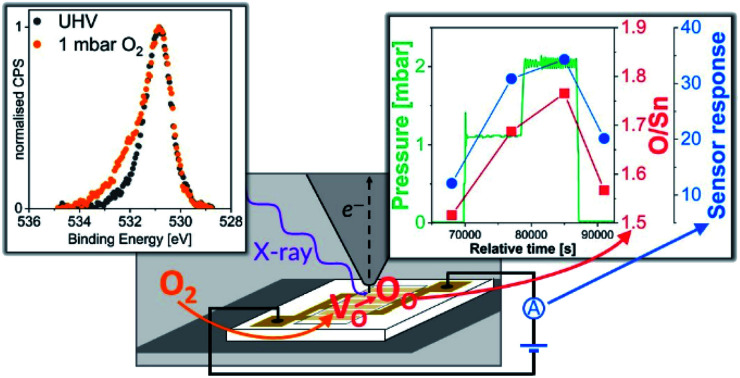

## Introduction

Conductometric gas sensors (CGS) typically provide a reproducible gas response, have low production and operation costs, and are sold in large quantities across the globe.^1^ However, CGS typically suffer from limited selectivity and long recovery times.^2^ The lack of significant progress in addressing these drawbacks can in part be ascribed to an incomplete picture of atomic interactions during sensing (‘receptor’ function) and their link with the measured macroscopic changes in materials properties (‘transducer’ function).^1^

Current models of gas sensitivity are based on macroscopic and ex situ spectroscopic measurements.^3^ The most commonly described is the oxygen ionosorption model, in which monoatomic O^−^ adsorbates are produced by dissociative adsorption of gaseous O_2_, trapping electrons from the conduction band (for an n-type semiconductor) at the material's surface.^4–8^ The resulting accumulation of negative surface charge induces an ‘electron depletion layer’ in the material, a volume of decreased Fermi energy (relative to the bulk value in the absence of surface charge). This causes upward band bending (relative to the surface in the absence of adsorbates – the ‘flat band’ condition), which is witnessed as increased sensor resistance.^9^ For the prototype CGS material, SnO_2_, there is no direct evidence for O^−^ species,^10^ and whilst other adsorbates have been shown to exist on SnO_2_, for example, O_2_^−^, their effect on resistance is considered small.^11^

**Fig. 1:**
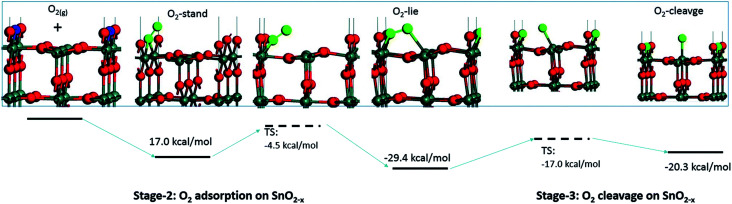
Visualisation of possible oxygen species adsorbed onto a surface V_O_ on the (110) surface of SnO_2_. The V_O_ (the locations of absent O atoms) are marked in blue. Reproduced with permission from ref. 12.

Computational studies have been used to provide new insights on the mechanism of interaction between gaseous species and the surface of sensing materials; in general such studies predict that O_2_ does not interact with stoichiometric surfaces of SnO_2_, highlighting oxygen vacancies (V_O_) as essential in enabling adsorption.^10,12–16^ These studies indicate that gaseous O_2_ adsorbed on a metal oxide dissociates in a thermally activated process (Fig. 1). However, this does not necessarily lead to the formation of ionosorbed O^−^; dissociation of O_2_ across a pair of surface *V*_O_ will lead to healing of both defects (removing adsorption sites in the process), with no ‘adsorbates’ remaining on the surface (*i.e.* reversible binding of oxygen at a lattice site).^12–14^

The consideration of these V_O_ ‘active sites’ significantly affects the description of band bending at the surface. V_O_ in their neutral state contain two electrons; their formation in the vicinity of the surface introduces donor states below the conduction band minimum in the near-surface region. The ionisation of surface V_O_ (*e.g.* at sufficiently high temperature) must cause accumulation of positive surface charge, inducing a surface ‘electron accumulation layer’, a volume of increased Fermi energy (relative to the bulk value in the absence of surface oxygen vacancies). This causes downward band bending at the surface (relative to the surface in the absence of surface oxygen vacancies – the ‘flat band’ condition). Therefore, the healing of V_O_ with increasing oxygen partial pressure is expected to lead to (relatively) upward band bending as a function of decreasing oxygen vacancy concentration. It is worth noting that upward band bending and increased resistance is expected in both ionosorption (increased surface adsorbates) and vacancy modulation (decreased oxygen vacancy concentration) descriptions with increasing oxygen partial pressure, *i.e.* they are synonymous at the macroscopic scale.

Using ex situ X-ray photoelectron spectroscopy measurements (XPS), Semancik *et al.* have previously inferred a link between decreasing surface V_O_ concentration and increased resistance in SnO_2_,^15^ and have also shown that the formation of surface V_O_ results in a surface conductivity layer, and additionally an alternative gas sensing mechanism employing a description of ionised surface oxygen vacancies has recently been proposed.^16^ Further, Elger and Hess recently published a study correlating changes in the ambient gas composition with surface adsorbed species, inferring changes in oxygen vacancy concentration from UV-vis reflection spectra.^17^

However, to directly link variation in vacancy concentration with change in materials resistance as a function of gas ambient requires a joint macroscopic and spectroscopic picture of a sensor working dynamically under typical temperature and pressure operating conditions. Such ‘operando’ investigations have previously been reported for a variety of spectroscopic techniques.^18^ Near-ambient pressure (NAP) XPS allows investigation of the chemical state and relative abundance of a material's near-surface atoms through representative spectral peaks in pressures up to several millibars,^19^ and also provides information on band bending through shifts in the binding energy (BE) scale, and consequently it has started to find application for studying sensors under conditions close to their normal operating environment.^20–22^ However, to date, there has been no extensive investigation of the most fundamental gas/surface interaction in SnO_2_ gas sensors, that with O_2_, and in particular, no in-depth study of oxygen vacancies under dynamic conditions has been presented. In this paper, we use NAP XPS to elucidate the dynamic effect of O_2_ adsorption on the electronic properties of SnO_2_ highlighting the role of oxygen vacancies and their interactions with O_2_ adsorbates in the gas sensing mechanism of SnO_2_.

## Results and discussion

This investigation aimed to examine oxygen adsorption on polycrystalline SnO_2_ (as used in the majority of CGS) without extrinsic dopants but with an appreciable density of surface vacancies. Such a surface was achieved through *in situ* H_2_ reduction and dehydroxylation immediately prior to the experiments at the ‘VerSoX’ beamline B07 of Diamond Light Source (for details, see ref. 23 and ESI†). This paper presents two experiments: LOW_T, performed at 50 °C, and HIGH_T, performed at 350 °C. The two temperatures were chosen to represent conditions below and above the 150 °C threshold at which, according to ‘ionosorption’ models, diatomic ionosorbed oxygen species (*e.g.* O_2_^−^) give way to monoatomic ones (*e.g.* O^−^).^6^ After establishing a constant temperature, the pressure in the analysis chamber was altered according to the planned experimental steps, denoted as D# (dosing of 1 or 2 mbar O_2_) and E# (evacuation to UHV). The sensor's resistance was monitored continuously throughout each experiment, including during XPS acquisition, and the XPS measurements were collected after every pressure change (allowing approximately 1 hour for sample stabilisation); no measurable changes in the resistance were observed as a result of X-ray irradiation of the sensor. The tuneable excitation energy of synchrotron-based XPS was used to examine the spatial distribution of the observed species. The two photon energies used (895 eV and 2000 eV) resulted in sampling depths of approximately 2.5 nm and 5 nm, referred to as ‘shallow’ and ‘deep’, respectively, in this text (see ESI† for depth estimation details). Given the experimental uncertainties, the values of parameters (band bending, O/Sn and ‘O third/Sn’) derived from the XPS spectra reported below have been rounded to the nearest 0.05.

### Low-temperature experiment (LOW_T)

**Fig. 2:**
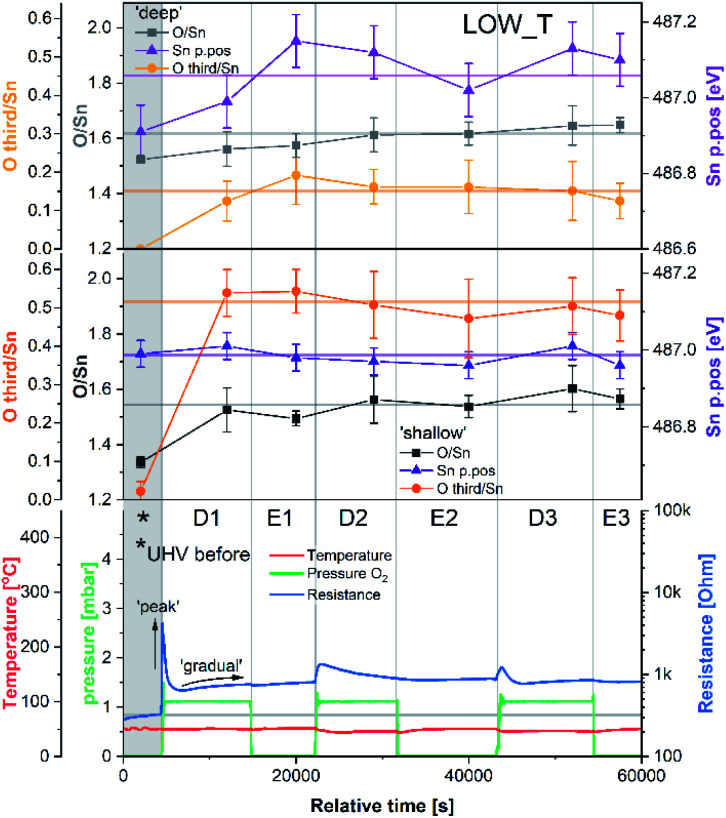
Results of phenomenological (bottom) and XPS (middle and top) investigation during experiment LOW_T. The ‘shallow’ and ‘deep’ plots present quantification of XPS spectra with 2.5 and 5 nm sampling depth, respectively. Sn p.pos denotes the binding energy position of the Sn 3d_5/2_ peak.

The results of the analysis during LOW_T are presented in Fig. 2. Starting with macroscopic data (bottom panel of Fig. 2), the resistance of the sensor after reduction (indicated on the figure as ‘UHV before’) stabilised around 300 Ω (for details on the reduction procedure, see ESI†). As soon as O_2_ was introduced (D1), the resistance increased rapidly up to 4.2 kΩ before immediately starting to decrease to 640 Ω. This phenomenon happened before the O_2_ pressure reached the maximum value (see Fig. S8 in ESI†) and therefore is not linked to the observed overshoot in O_2_ pressure and subsequent stabilisation at 1 mbar. This initial ‘peak-type’ response was followed by a gradual, small increase and stabilisation of the resistance at a value slightly larger than the initial one, about 750 Ω (response 2.5, *R*_present_/*R*_previous_). Notably, the sensor's resistance was insensitive to the subsequent O_2_ evacuation (E1), and there was no decrease in resistance upon evacuation during any of the E# steps. Each following oxygen exposure after D1 had a smaller effect on both the ‘peak’ and ‘gradual’ response; after stabilisation in D2, the resistance was around 850 Ω (an increase of 100 Ω compared to D1, *i.e.* a response of 1.1) and after stabilisation in D3, the resistance was at the same value as in D2 (850 Ω, *i.e.* a response of 1.0). Together, these results indicate that, from the macroscopic point of view, the changes occurring at the surface of SnO_2_ upon exposure to O_2_ at low temperature (50 °C) are largely irreversible. The limited change in resistance observed from D1 onwards means that the microscopic processes occurring here cannot be the ones responsible for the high sensitivity of SnO_2_-based sensors (the invariance of resistance measurements over an extended time period for each dose suggests the limited change in resistance is not simply a function of slow kinetics but is indicative of a ‘saturated’ response).

**Fig. 3:**
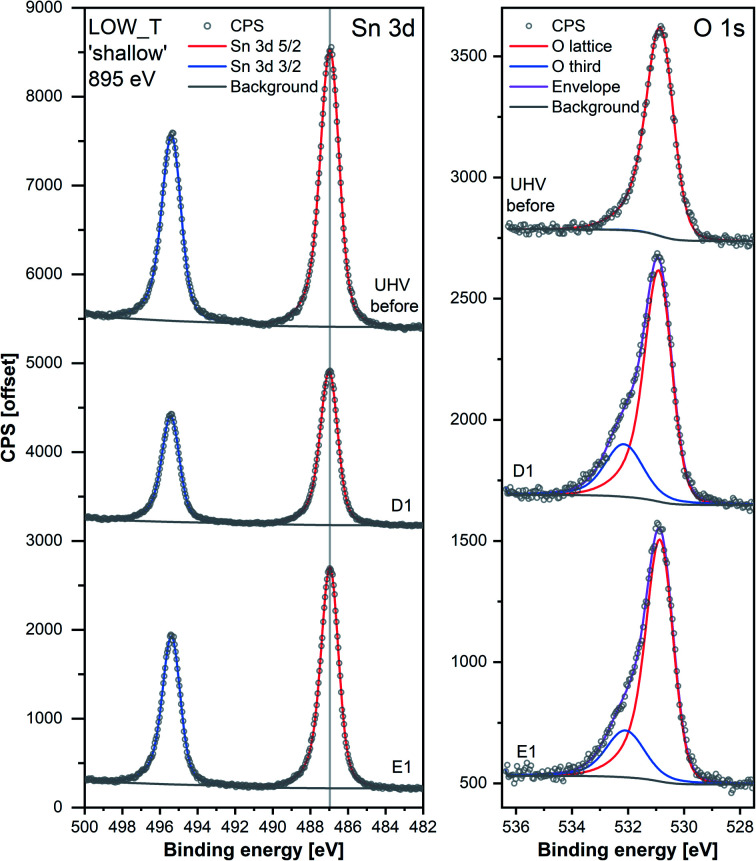
Comparison of Sn 3d and O 1s high-resolution spectra collected during steps ‘UHV before’, D1 and E1 of experiment LOW_T. Peaks were offset along the *Y*-axis. The lower signal intensity of Sn during D1 compared to the UHV steps is attributable to the attenuation of photoelectrons by the O_2_ gas present during that step. The O 1s peaks were normalised with respect to their corresponding Sn 3d peak areas.

Changes in the corresponding XPS spectra were used to understand the microscopic processes occurring at the sensor's surface during each dose and evacuation step. The Sn 3d spectra collected during LOW_T (Fig. 3) showed no changes in the peak shape resulting from O_2_ exposure. The well-defined shape of the Sn peaks allows reliable estimation of their position and, in turn, band bending analysis, which is presented in Fig. 2 (middle panel, Sn p.pos). After reaching the experimental temperature (‘UHV before’), the ‘shallow’ Sn 3d peak appeared at 487.00 eV, and no O_2_ pressure-dependent changes in its position were observed irrespective of the dose or evacuation state. Likewise, in the ‘deep’ spectra, no peak shifts were discernible beyond experimental uncertainty. Therefore we conclude that on exposure of the sensor to O_2_ at 50 °C there is no observable band bending.

Despite the fact that no band bending was observed, the dosing of O_2_ significantly changed the peak shape of the O 1s photoemission. In order to assess these changes quantitatively, the peak was fitted with two components (for details, see ESI†). The first component (denoted ‘O lattice’ in Fig. 3) corresponds to the O atoms constituting SnO_2_ and was fitted without any constraints in its position, FWHM or area. After introducing O_2_ (D1), another peak was required to obtain a good fit, corresponding to newly-formed oxygen species, arbitrarily denoted as ‘O third’ to indicate that they are not related to the SnO_2_ lattice, *i.e.* ‘third-party species’. The ‘O third’ component parameters (position, FWHM and area) were also left unconstrained, and their magnitude is a measure of the difference between the shapes of the ‘O lattice’ component and the observed photoemission. Similar peak fitting was performed at every step of the investigation for the ‘shallow’ and ‘deep’ spectra, allowing estimation of the relative abundance and distribution of ‘O lattice’ and ‘O third’, reported as O/Sn and ‘O third’/Sn ratios.

The quantification described above is presented in Fig. 2 over multiple dose and evacuation steps and shows that the ‘O third’/Sn ratio increased during D1 (1 mbar O_2_) from approximately 0 during ‘UHV before’ for both ‘shallow’ and ‘deep’ spectra to 0.50 for shallow spectra (middle panel) and 0.15 for deep spectra (top panel). Subsequently, neither the removal (E#) nor reintroduction (D#) of O_2_ influenced the ‘O third’ peak intensity, which remained constant within experimental precision. The larger increase in the ‘shallow’ (0.50) compared to ‘deep’ (0.15) spectra indicates that the new oxygen species are localised close to the surface. However, relatively little information about the surface species’ chemical identity can be extracted from these spectra (Fig. 3). The position of ‘O third’, *ca.* 532.10 eV, falls within a binding energy region characteristic for many species, including adventitious organic contamination, surface hydroxyls and adsorbed water, as well as for oxygen adsorbates.^20,24^ Therefore, the identity of the species present cannot be determined from the binding energy of the peak alone but has to be deduced *via* exclusion analysis. Considering the high purity O_2_ used in this experiment (N6.0, H_2_ ≤ 0.05 ppm, H_2_O ≤ 0.5 ppm) and the mismatch between trends observed in ‘O third’ and C 1s spectra (which, if correlated, would point to the ‘O third’ emission originating from organic contamination), it is concluded that the species appearing at the surface were not related to surface hydroxylation or to adventitious organic contamination. For detailed considerations, see ESI.†

Since other possibilities were excluded, the ‘O third’ peak must be ascribed to some form of adsorbed oxygen species. However, these are unlikely to be the O^−^ species invoked in the ionosorption model of gas sensing, as the surface density of such species is limited to 10^−3^–10^−5^ of a monolayer.^11^ Assuming that only the outermost surface atoms contribute to the signal (3 O and 2 Sn atoms per (110) surface unit cell) and the maximum adsorbate density (10^−3^ ML; 2 O^−^ per unit cell is 1 ML), the O^−^ adsorbate peak should be no larger than 0.13% of the lattice oxygen peak area, and even smaller when all lattice atoms in the sampling depth are considered. This is much less than the area of ‘O third’ relative to ‘O lattice’ (35%) and below the detection limit in XPS.^25^ Therefore, given their large relative abundance the species must be uncharged with respect to the lattice and therefore likely to be diatomic and covalently bound onto surface V_O_ (*i.e.* species identified as O_2_-stand and O_2_-lie in Fig. 1). After the initial increase between ‘UHV before’ and D1, indicating the appearance of adsorbates, the ‘O third’/Sn ratio (Fig. 2) did not decrease significantly upon evacuation in any of the following E# steps. This means that at 50 °C adsorption is irreversible, and the adsorption sites are not recovered in UHV. Since the ratio reaches its plateau value during D1, it can be assumed that nearly all active adsorption sites are occupied at this point; consequently, the lack of additional available sites prevents the adsorption of new dioxygen molecules during D2 and D3. Finally, the formation of these oxygen adsorbates does not influence band bending, as the Sn p.pos does not change when ‘O third’ emerges (Fig. 2, middle panel). Therefore, these adsorbates are not connected to the sensor's response as normally described under the framework of ionosorption theory, and other mechanisms for resistance change must be at play.

The area of the ‘O lattice’ component (Fig. 3) divided by the corresponding Sn 3d peaks, denoted as O/Sn ratio, determines the SnO_2−*x*_ lattice stoichiometry and therefore measures the V_O_ density in the analysed volume. In the ‘shallow’ spectra (Fig. 2, middle panel), the O/Sn ratio was approximately 1.35 after reduction (‘UHV before’). During steps D1 and E1, the ratio increased to 1.50 to 1.60 and subsequently remained invariant within experimental precision (note these values are calculated using standard relative sensitivity factors and therefore, whilst comparable with one another, do not represent the true Sn:O stoichiometry). At the same time, the O/Sn ratio derived from ‘deep’ spectra (Fig. 2, top panel) showed a steady, gradual increase from 1.50 to 1.60 throughout the experiment and, considering the uncertainties, is barely noticeable. Since this parameter determines the SnO_2_ stoichiometry in the analysed volume, an increase can be interpreted as a measure of oxygen vacancy healing *via* an influx of oxygen into the lattice. The larger variation in ‘shallow’ O/Sn suggests that the stoichiometry change is primarily occurring very close to the surface, consistent with dynamic exchange of oxygen atoms with surface vacancies. Given that the changes in the ‘shallow’ volume will also affect the ‘deep’ spectra, and considering the low rate of V_O_ hopping in SnO_2_ at 50 °C (very slow diffusion),^26,27^ it is likely that the ‘deep’ O/Sn increase is entirely due to the near-surface stoichiometry change observed in ‘shallow’ spectra.

These trends in the O/Sn ratio correlate with the small, gradual overall increase in resistance observed during LOW_T. The largest increase in both the O/Sn ratio (1.35 to 1.50, Fig. 2, middle panel) and the resistance (300 Ω to 750 Ω, Fig. 2, bottom panel) is observed (for measurements made in UHV) after the first O_2_ exposure (‘UHV before’ *vs.* E1). Subsequently, both O/Sn and resistance increase less between E1 and E2 and negligibly between E2 and E3. Moreover, the temperature of 50 °C is considered too low to enable spontaneous surface reduction; therefore, neither the resistance nor the O/Sn ratio decrease in UHV but progress towards their equilibrium values determined by the oxygen pressure during exposure.

**Fig. 4:**
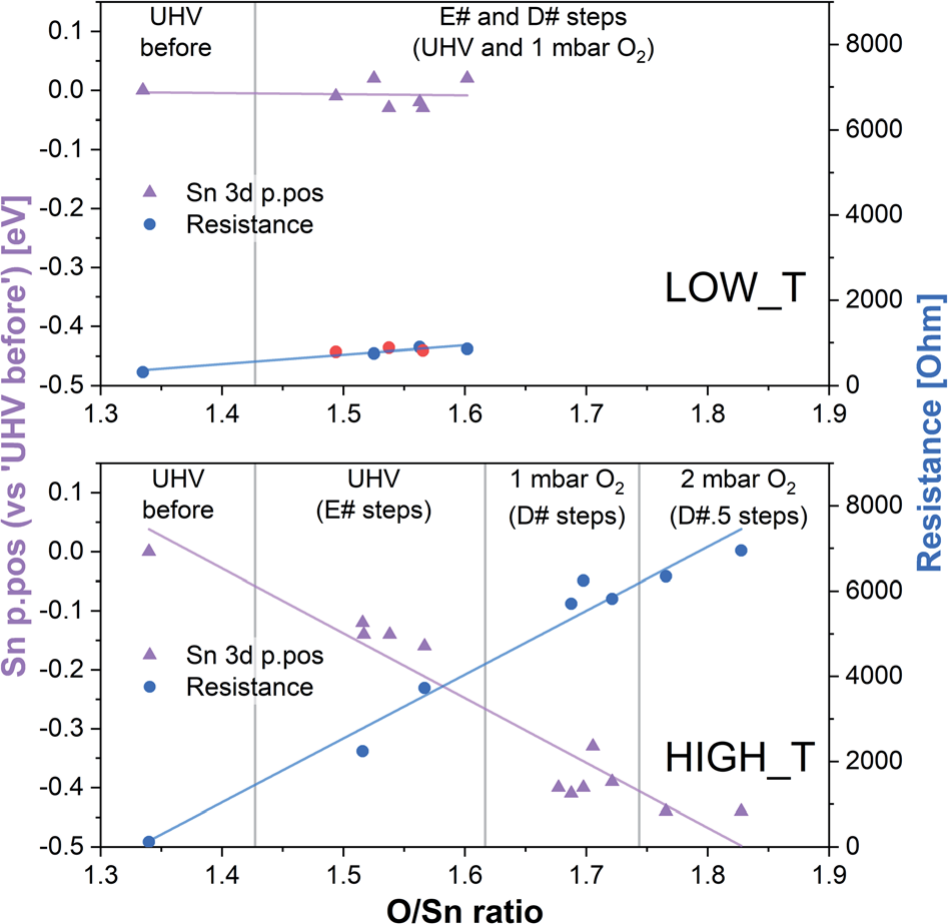
Summary of band bending (Sn 3d p.pos) and resistance change as a function of oxygen vacancy density in the near surface region derived from the LOW_T and HIGH_T experiments. In the LOW_T plot, the red resistance data points correspond to E# steps. In the HIGH_T plot, the ‘anomalous’ resistance data were omitted; for details, see the discussion of the HIGH_T experiment below and ESI†.

To try and describe the features seen in LOW_T, we refer to the model by Yang *et al.* (Fig. 1). We conjecture that after H_2_ reduction and dehydroxylation, the surface is vacancy rich (low O/Sn ratio), and on initial O_2_ exposure, the O_2_-stand configuration of adsorbed oxygen is obtained (‘O third’), causing the initial peak change in resistance. Subsequently, the O_2_-stand is converted to the O_2_-lie configuration, correlating with the resistance drop immediately after the peak. This is not entirely consistent with the description of Yang, as the partial charge (charge transferred from SnO_2_ to O_2_) of O_2_-lie is calculated to be greater than for O_2_-stand, however a change in nature of adsorbed species such as this would account for the resistance profile observed. These surface species are clearly stable at 50 °C as exposure to UHV does not cause the species to desorb (cycling between D# and E# does not change the ‘O third’/Sn ratio). We also conjecture that at this temperature (compared to 350 °C, see below), there is not enough energy to overcome the barrier to dissociate all the adsorbed oxygen fully (Fig. 1). The dissociation of O_2_ corresponds to the maximum charge transfer observed by Yang and hence is expected to correspond to a large change in sensor resistance. However, this provides an inconsistent piece of data – the change in ‘lattice oxygen’ (O/Sn) at 50 °C is significant (1.35 to 1.50) but does not correlate with significant resistance change (compared to 350 °C, see Fig. 4 and discussion below). Referring to the recently described surface vacancy description of gas sensor response,^16^ a potential explanation for this lack of resistance change is that the number of ionised near-surface oxygen vacancies increases with increasing temperature;^28^ only ionised V_O_ are expected to contribute to resistance change (non-ionised neutral V_O_ already localise electrons at the vacancy site). This would also account for the lack of band bending observed even though the vacancy concentration appears to vary significantly.

In summary of the LOW_T experiment, we observed a substantial amount of oxygen adsorbates which, due to the lack of corresponding band bending, are likely diatomic and not (formally) charged (see Fig. 1), and not directly related to the response generation in SnO_2_. At this temperature, the O_2_ adsorption is irreversible since ‘O third’ does not decrease in UHV. Moreover, the lack of bend bending and minimal resistance change with increasing surface stoichiometry, as presented in Fig. 4, suggest that adsorbate dissociation does not localise electrons onto non-ionised vacancies.^16^ Additionally, given that the low temperature prevents vacancy diffusion from the bulk towards the surface, any vacancies healed here must be limited to the surface-most atomic layer, which is reflected in the significant difference between ‘shallow’ and ‘deep’ O/Sn variance. Finally, the link between the observed decrease in vacancy density and baseline resistance increase through ‘gradual’ response is consistent with other studies, in which changing oxygen vacancy concentration was correlated with sensor drift.^26^

### High-temperature experiment (HIGH_T)

**Fig. 5:**
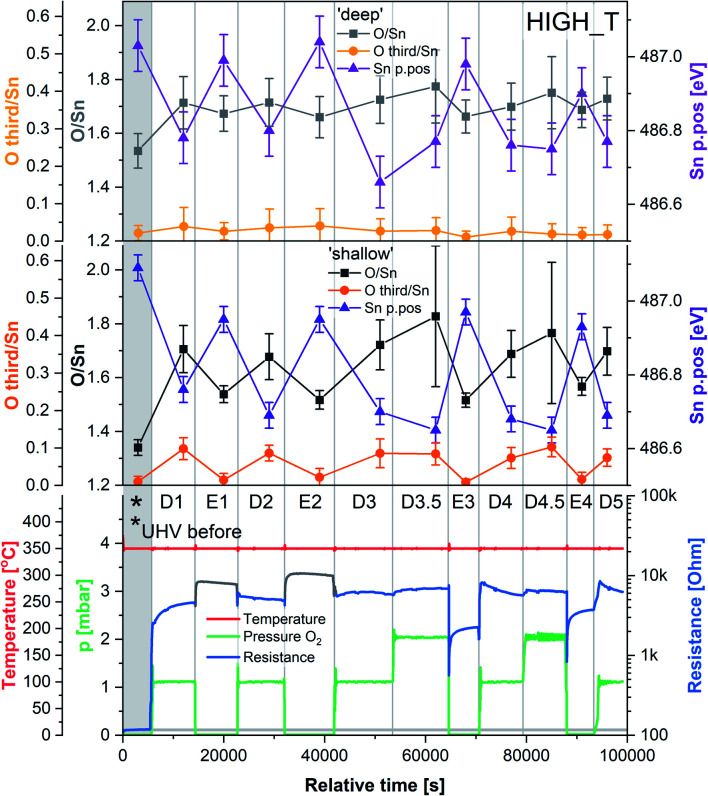
Results of phenomenological (bottom) and XPS (middle and top) investigation during experiment HIGH_T. The ‘shallow’ and ‘deep’ plots present quantification of XPS spectra with 2.5 and 5 nm sampling depth, respectively. Sn p.pos denotes the binding energy position of the Sn 3d_5/2_ peak.

The results of the HIGH_T experiment, analogous to LOW_T but performed at 350 °C, are presented in Fig. 5. Similar to LOW_T, the lowest resistance (120 Ω, ‘UHV before’) was observed directly after reduction (for details, see ESI†). During D1, the resistance increased quickly to 2.5 kΩ and then gradually to 4.5 kΩ (response 38, substantially higher than the LOW_T response of 2.5). The resistance measurements during E1 and E2 (UHV) are considered anomalous as resistance increased on evacuation, and therefore they have been greyed out in Fig. 5 (see ESI†). This behaviour was observed during the live experiment, and consequently, the O_2_ dose pressure was increased to 2 mbar (D3.5, D4.5), at which point the sensor started behaving as expected, *i.e.* resistance decreased on evacuation to UHV. The pressure increase during D3.5 caused the resistance to rise to approximately 7 kΩ; however, unlike in LOW_T, the subsequent removal of O_2_ (E3) resulted in a pronounced decrease in resistance (initially down to 550 Ω although later only to 2.3 kΩ once the resistance value stabilised). Reproducible changes in resistance were observed during subsequent steps, with the resistance increasing to around 6–7 kΩ during exposure to O_2_ (D4, D4.5 and D5) and decreasing to 3.5 kΩ after stabilisation in E4 (Fig. 5).

**Fig. 6:**
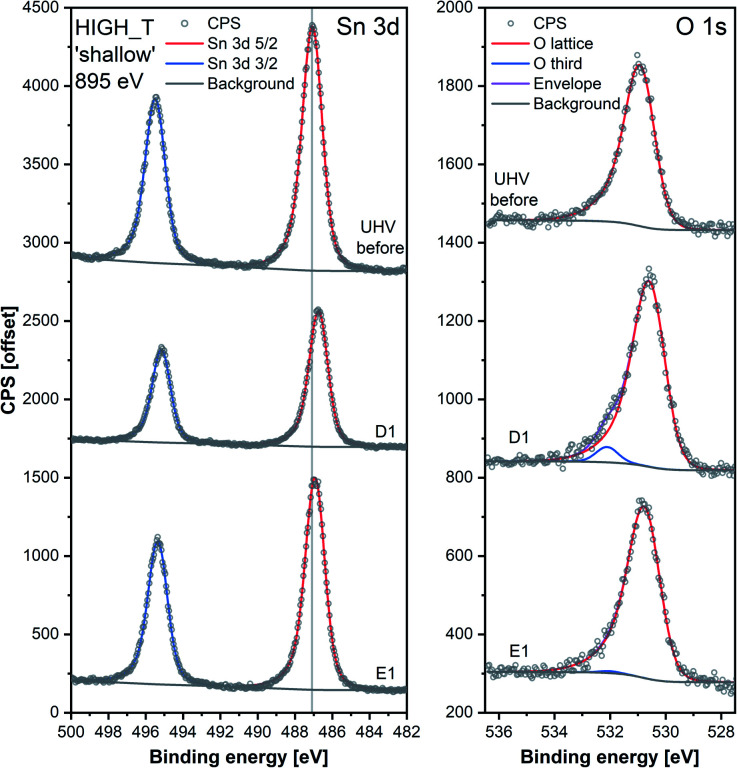
Comparison of Sn 3d and O 1s high-resolution spectra collected during steps ‘UHV before’, D1 and E1 of experiment HIGH_T. Peaks were offset along the *Y*-axis. The lower signal intensity of Sn during D1 compared to the UHV steps is attributable to the attenuation of photoelectrons by the O_2_ gas present during that step. The O 1s peaks were normalised with respect to their corresponding Sn 3d peak areas.

The ‘shallow’ band bending analysis (Sn p.pos, Fig. 5, middle panel) is derived from the Sn 3d spectra shown in Fig. 6. Significant oxygen pressure-dependent changes in band bending were observed during HIGH_T in the ‘shallow’ spectra, in contrast to LOW_T (Fig. 2) where negligible band bending was observed. In D1, the Sn peak shifts from 487.10 eV to 486.75 eV due to the bands bending upwards by over 0.30 eV. The subsequent O_2_ removal (E1) caused unbending of the bands; however, the Sn p.pos did not revert to its original value of 487.10 eV, but only to 486.95 eV, a value closer to that before reduction (487.00 eV). This value became the new maximum, to which the peak returned during subsequent E# steps. During the D# steps, the Sn p.pos consistently shifted to 486.70 eV (−0.25 eV), apart from D3.5 and D4.5, during which it was slightly lower at around 486.65 eV (−0.30 eV), correlating with the higher O_2_ pressure used during these steps. Similar trends were observed in ‘deep’ spectra (Fig. 5, top panel), although the values were less consistent due to the larger uncertainty associated with higher photon energy used to obtain the data (see band bending discussion in LOW_T). Additionally, the difference between Sn p.pos during D# and E# was smaller in ‘deep’ spectra, varying by only about 0.20 eV (between 486.80 and 487.00 eV) as opposed to 0.30 eV in ‘shallow’ spectra. This is consistent with the bands bending more strongly close to the surface, with the ‘deep’ Sn 3d peak position influenced more by the constant bulk Fermi level and the ‘shallow’ peak influenced more by the surface Fermi energy pinning.

The O/Sn ratio also showed remarkable reversibility in the ‘shallow’ spectra (Fig. 5, middle panel), as seen for band bending. Starting at an O/Sn value of 1.35 after reduction (the same as seen in LOW_T), the ratio increased significantly in D1 to about 1.70 (1.50 in LOW_T). Unlike LOW_T, O_2_ evacuation (E1) resulted in a decrease in O/Sn stoichiometry (beyond experimental uncertainty), down to 1.55 (*i.e.* not as ‘reduced’ as the starting point after H_2_ reduction). The O/Sn values of 1.70 for D# and 1.55 for E# were then consistently reproduced in all steps, except D3.5 and D4.5, where the O/Sn ratio was slightly larger (approximately 1.80), consistent with the increased O_2_ pressure (although it is worth noting that the D3.5 and D4.5 values carry a larger uncertainty in the O/Sn ratio due to the increased O_2_ pressure during data collection and the increased uncertainty in the required signal attenuation calibration, see ESI†).

Although similar trends were observed in the ‘deep’ O/Sn ratio (Fig. 5, top panel), the changes were less pronounced, similar to observations in LOW_T, with the initial O/Sn value of about 1.50 (‘UHV before’) increasing to 1.70 on oxygen dosing (D1). Upon removal of O_2_, the O/Sn value in the deep spectra decreased by about 0.05 and oscillated between 1.70–1.75 during D# steps and 1.65–1.70 during E# steps. The error bars associated with these values are significantly overlapping, indicating that the observed changes (apart from the initial increase in D1) are within uncertainty.

The difference between the lattice O/Sn ratio in ‘shallow’ and ‘deep’ spectra (middle and top panels respectively, Fig. 5) under UHV conditions suggests that oxygen vacancies are dynamically formed mainly close to the surface. In the ‘shallow’ spectra, the O/Sn ratio increased by 26% (1.70/1.35) upon O_2_ exposure in step D1. This same change resulted in only a 13% (1.70/1.50) increase in the ‘deep’ O/Sn ratio. Subsequent O_2_ evacuation in E1 caused a 9% (1.55/1.70) decrease in the ‘shallow’ spectra and only about 3% (1.65/1.70) in the ‘deep’. If the V_O_ were distributed homogenously beyond the thickness of the ‘shallow’ analysis volume, identical changes would be expected in both sets of spectra, hence it can be concluded that the V_O_ (healed or produced, depending on the conditions) constituted a more significant fraction of the lattice sites at the surface.

The variable density of near-surface oxygen vacancies, which reflects changes in the O_2_ pressure, is correlated with the trends in both the band bending (Sn p.pos) and sensor resistance, as shown in Fig. 4 (HIGH_T). As the density of vacancies decreases due to the incorporation of gaseous O_2_ into the lattice, the bands bend upwards (Sn p.pos decreases) and the resistance of the sensor increases. The changes are reproducible, since the repeated exposure to O_2_ and evacuation to UHV return the system to a similar *V*_O_ density, band bending and resistance as during the previous analogous steps, thus allowing distinction of separate regions on the plot corresponding to the E#, D# and D#.5 steps (in addition to the ‘UHV before’ which can only be attained through *in situ* reduction in H_2_). The close correlation between these three parameters indicate that the charge carrier concentration, which determines the Fermi level position and hence observable band bending and sensor's conductivity, depends on the density of surface vacancies, *i.e.* the vacancies act as electron donors, directly determining the sensor's resistance. A conductivity model based on vacancy healing and formation was recently proposed for SnO_2_-based CGS by Sun and Lu, which allowed them to model the conduction electron concentration and sensor response in both the ‘electron depletion’ and ‘electron accumulation’ regime depending on the density of near-surface vacancies relative to the bulk value.^29^ Our direct *in situ* spectroscopic evidence of the correlation between near-surface vacancy density and sensor's resistance and band bending fully supports such a mechanism.

Whereas an abundance of adsorbates was observed in the XPS spectra during LOW_T (Fig. 3), the sensor's exposure to O_2_ in HIGH_T has relatively little effect on the shape of the O 1s photoemission spectra; Fig. 6 shows the changes in ‘shallow’ O 1s peaks between ‘UHV before’, D1 and E1. The binding energy position of the lattice component shifts visibly after O_2_ exposure (‘UHV before’ *vs.* D1 and E1), which is also visible in the Sn 3d peaks, and is indicative of band bending. Again, while a single component (‘O lattice’) is sufficient to fit the observed photoemission of ‘UHV before’, another component, ‘O third’, is required in the case of D1. However, the magnitude of this component (‘O third’/Sn ratio of 0.10) is only 6% of the lattice peak, which is relatively small compared to the analogous peaks in LOW_T (35%). Given that the peak appears as a result of O_2_ exposure and at the same binding energy as during the LOW_T experiment, *ca.* 532.10 eV, again consistent with the ‘O third’ species being oxygen adsorbates,^24^ it was therefore assigned to diatomic (formally) neutral oxygen adsorbed onto surface oxygen vacancies, such as the O_2_-stand and O_2_-lie species shown in Fig. 1. Notably, in contrast to LOW_T, the ‘O third’ emission for HIGH_T disappears after evacuation (E1 peak in Fig. 5), *i.e.* fully reversible formation of oxygen adsorbates is observed. As shown in Fig. 5 (‘O third’/Sn), the component reappears in all subsequent D# steps at a similar intensity and is removed during the following E# steps (‘O third’/Sn oscillates between circa 0.10 for D# and 0 for E#). While the change in ‘O third’ between D# and E# steps is evident in the ‘shallow’ spectra, the ‘deep’ spectra show much smaller variation (the error bars of data points overlap significantly, and there are no clear trends visible). Although the amount of detected oxygen adsorbates (‘O third’) in HIGH_T is substantially smaller than in LOW_T, it is still well above the theoretical maximum for charged adsorbates (Weisz limit, ‘O third’/‘O lattice’ ≤ 0.13%). Therefore, these spectroscopically observed species are unlikely to be O^−^, even though the temperature of this experiment has previously been associated with O_2_ dissociation into charged monoatomic species.^3^

The difference in ‘O third’ intensity between the LOW_T and HIGH_T experiments can be rationalised in terms of two factors. Firstly, the elevated temperatures cause increased dissociation of the adsorbed dioxygen, healing one or two oxygen vacancies and removing adsorption sites in the process.^15^ Supporting this notion is the observed increase in the O/Sn ratio during O_2_ exposures (D# in Fig. 5), more pronounced in the ‘shallow’ spectra, indicating surface vacancy healing and (coincidentally) removing some adsorption sites. Secondly, since the ‘O third’ peak disappears in UHV, it can be concluded that the adsorption of O_2_ is reversible at this temperature, in agreement with TPD and ESR studies which indicate that diatomic oxygen desorbs from the surface by around 100–150 °C,^11^ suggesting a more dynamic occupation of available adsorption sites than at low temperature.

**Fig. 7:**
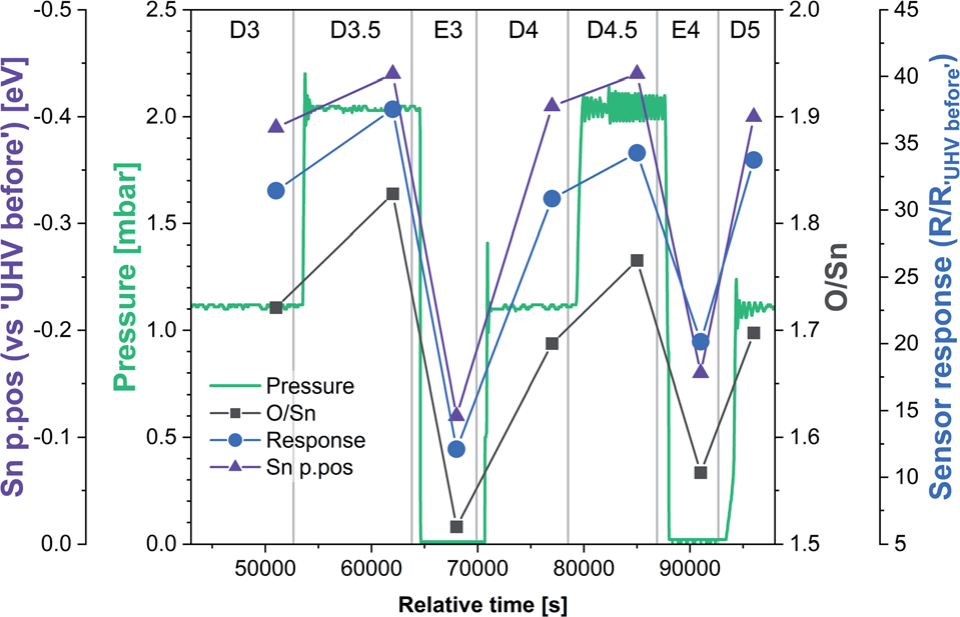
Correlation between O/Sn ratio of the lattice component (V_O_ concentration), Sn p.pos relative to ‘UHV before’ (band bending) and the sensor response under dynamically changing O_2_ pressure conditions during final stages of experiment HIGH_T. The initial steps were omitted due to the anomalous resistance measurements recorded during steps E1 and E2.

Although we cannot exclude the presence of low concentrations of charged oxygen adsorbates below the detection limit of our experiment, it is clear that the surface oxygen vacancy concentration changes very significantly when cycling between UHV and 1 mbar O_2_ at 350 °C (around a 25% change in surface lattice oxygen concentration – O/Sn ratio). These changes in V_O_ concentration (Fig. 7, O/Sn) must be expected to exert a large influence on the electronic structure (charge distribution) in the near-surface region (assuming surface *V*_O_ are ionised at the given temperature) and are correlated with both band bending and sensor response (Fig. 7) as a function of O_2_ pressure.^16^ Application of ‘Occam's Razor’ therefore leads us to conclude that variable surface vacancy concentration is responsible for the O_2_ sensing mechanism of SnO_2_, not extrinsic charged oxygen species. A similar conclusion was reached by Andrae *et al.* who used synchrotron NAP XPS to examine the interaction of O_2_ with donor-doped SrTiO_3_ to interpret (non-simultaneous) resistance measurements.^30^ There, the actor for band bending (and hence resistance change) was also concluded not to be due to charged oxygen adsorbates, but was due to (increasing) formation of a depletion layer (q.v. decreasing formation of an accumulation layer) with increasing O_2_ partial pressure, resulting from formation of negatively charged strontium cation vacancies (q.v. positively charged oxygen anion vacancies) in the SrTiO_3_ lattice due to precipitation of phase-separated SrO at the surface. Our results are also qualitatively consistent with the results of Semancik *et al.*, who previously correlated upward band bending with surface oxidation and vacancy healing.^31^

## Conclusions

In this study, joint macro- and spectroscopic analyses were performed using synchrotron NAP XPS with simultaneous resistance measurements on sensors exposed to O_2_ and UHV at both room and elevated temperatures. The results of this study show that exposure of (*in situ* reduced) sensors to O_2_ at elevated temperatures results in resistance and band bending changes, typical of SnO_2_-based sensors operation. These parameters are correlated with significant changes in the near-surface oxygen vacancy concentration under dynamic pressure conditions and we conclusively show that the adsorbed oxygen species observed upon O_2_ dosing are not responsible for the measured resistance change. The decrease in surface oxygen vacancy concentration on O_2_ dosing must lead to a decrease in positive charge in the near-surface region of SnO_2_, and hence upward band bending, and therefore we see no need to invoke additional entities (*e.g.* negatively charged ‘ionosorbed’ oxygen) to describe the same phenomenon. Consequently, we conclude that the resistance change in SnO_2_ sensors in response to O_2_ does not result from extrinsic negatively charged surface oxygen species but rather from the dynamic exchange of lattice oxygen with the gas ambient *via* the formation and healing of surface oxygen vacancies. This challenges the current unproven conceptions in the field of gas sensing^8^ but is fully consistent with the recent work in the field of solid-state physics on vacancy-induced surface conductivity layers in metal oxides.^16^

Understanding the importance of oxygen vacancies provides rules for targeted design of higher performance (n-type) gas sensing materials *via* the deliberate manipulation of surface oxygen vacancy concentration, for instance by surface modification with high valent oxyphilic species.^32^ The method we developed can be readily applied to study a range of sensor materials under exposure to various gases, which we have demonstrated can provide new fundamental understanding of the surface chemistry in CGS. In light of the evidence presented in this paper, we believe such models represent a promising direction towards a complete understanding of CGS operation and hence their performance enhancement.

## Data availability

The datasets supporting this article have been uploaded as part of the ESI.†

## Author contributions

S. K.: conceptualization, data curation, formal analysis, investigation, methodology, visualisation, writing – original draft; C. Blackman: conceptualization, formal analysis, funding acquisition, project administration, supervision, writing – original draft; P. F.: conceptualization, investigation; G. H. and A. S. W.: conceptualization, writing – review & editing; C. Byrne.: conceptualization, methodology; F. V.: conceptualization; J. A. C. and S. K. A.: resources; A. M. B.: writing – review & editing.

## Conflicts of interest

There are no conflicts to declare.

## Supplementary Material

SC-013-D2SC01738E-s001

## References

[cit1] Barsan N., Koziej D., Weimar U. (2007). Sens. Actuators, B.

[cit2] Yamazoe N. (2005). Sens. Actuators, B.

[cit3] Sahm T., Gurlo A., Bârsan N., Weimar U. (2006). Sens. Actuators, B.

[cit4] Morrison S. R. (1987). Sens. Actuators.

[cit5] Geistlinger H. (1993). Sens. Actuators, B.

[cit6] Barsan N., Weimar U. (2001). J. Electroceram..

[cit7] Ding J., McAvoy T. J., Cavicchi R. E., Semancik S. (2001). Sens. Actuators, B.

[cit8] Staerz A., Weimar U., Barsan N. (2022). Sens. Actuators, B.

[cit9] Bârsan N., Hübner M., Weimar U. (2011). Sens. Actuators, B.

[cit10] Kucharski S., Blackman C. (2021). Chemosensors.

[cit11] Gurlo A. (2006). ChemPhysChem.

[cit12] Lu Z., Ma D., Yang L., Wang X., Xu G., Yang Z. (2014). Phys. Chem. Chem. Phys..

[cit13] Ducéré J.-M., Hemeryck A., Estève A., Rouhani M. D., Landa G., Ménini P., Tropis C., Maisonnat A., Fau P., Chaudret B. (2012). J. Comput. Chem..

[cit14] Wang X., Qin H., Chen Y., Hu J. (2014). J. Phys. Chem. C.

[cit15] Cox D. F., Fryberger T. B., Semancik S. (1988). Phys. Rev. B: Condens. Matter Mater. Phys..

[cit16] Blackman C. (2021). ACS Sens..

[cit17] Elger A., Hess C. (2019). Angew. Chem., Int. Ed..

[cit18] Gurlo A., Riedel R. (2007). Angew. Chem., Int. Ed..

[cit19] Starr D. E., Liu Z., Hävecker M., Knop-Gericke A., Bluhm H. (2013). Chem. Soc. Rev..

[cit20] Vorokhta M., Khalakhan I., Vondráček M., Tomeček D., Vorokhta M., Marešová E., Nováková J., Vlček J., Fitl P., Novotný M., Hozák P., Lančok J., Vrňata M., Matolínová I., Matolín V. (2018). Surf. Sci..

[cit21] Hozák P., Vorokhta M., Khalakhan I., Jarkovská K., Cibulková J., Fitl P., Vlček J., Fara J., Tomeček D., Novotný M., Vorokhta M., Lančok J., Matolínová I., Vrňata M. (2019). J. Phys. Chem. C.

[cit22] Junker B., Favaro M., Starr D. E., Hävecker M., Weimar U., Barsan N. (2022). J. Phys. D: Appl. Phys..

[cit23] Held G., Venturini F., Grinter D. C., Ferrer P., Arrigo R., Deacon L., Quevedo Garzon W., Roy K., Large A., Stephens C., Watts A., Larkin P., Hand M., Wang H., Pratt L., Mudd J. J., Richardson T., Patel S., Hillman M., Scott S. (2020). J. Synchrotron Radiat..

[cit24] Ciftyürek E., Šmíd B., Li Z., Matolín V., Schierbaum K. (2019). Sensors.

[cit25] Shard A. G. (2014). Surf. Interface Anal..

[cit26] Kamp B., Merkle R., Lauck R., Maier J. (2005). J. Solid State Chem..

[cit27] De Frésart E., Darville J., Gilles J. M. (1982). Appl. Surf. Sci..

[cit28] Maier J., Göpel W. (1988). J. Solid State Chem..

[cit29] Zhao L., Gong X., Tao W., Wang T., Sun P., Liu F., Liang X., Liu F., Wang Y., Lu G. (2022). ACS Sens..

[cit30] Andrä M., Bluhm H., Dittmann R., Schneider C. M., Waser R., Mueller D. N., Gunkel F. (2019). Phys. Rev. Mater..

[cit31] Cavicchi R., Tarlov M., Semancik S. (1990). J. Vac. Sci. Technol., A.

[cit32] Dai J., Frantzeskakis E., Fortuna F., Lömker P., Yukawa R., Thees M., Sengupta S., Le Fèvre P., Bertran F., Rault J. E., Horiba K., Müller M., Kumigashira H., Santander-Syro A. F. (2020). Phys. Rev. B.

