# Supplementary material for: Direct in situ spectroscopic evidence of the crucial role played by surface oxygen vacancies in the O2-sensing mechanism of SnO2
Source: Chem Sci. 2022 May 5;13(20):6089–97. doi: 10.1039/d2sc01738e (PMC9132051; doi:10.1039/d2sc01738e)
Supplement: SC-013-D2SC01738E-s001 [file SC-013-D2SC01738E-s001.pdf]

Electronic Supplementary Material (ESI) for Chemical Science.
This journal is © The Royal Society of Chemistry 2022

Electronic Supplementary Information (ESI)

Direct in situ spectroscopic evidence of the crucial role played by surface oxygen vacancies in the O_2-sensing mechanism of SnO_2

S. Kucharski, P. Ferrer, F. Venturini, G. Held, A. S. Walton, C. Byrne, J. A. Covington, S. K. Ayyala, A. M. Beale and C. Blackman*

Contents

Experimental Section 2
- Sample sensor preparation 2
- Instruments used 2
- Data acquisition procedure 3
- Signal attenuation by gas under NAP conditions 5
- Sampling depth estimation 6
- Spectra analysis 8

LOW_T 11
- In situ reduction 11
- Sn 3d and O 1s fit parameters for LOW_T 13
- Considerations on the origin of 'O third' in LOW_T during O_2 dosing 14
- Magnification of D1 resistance change 17

HIGH_T 18
- In situ reduction 18
- Sn 3d and O 1s fit parameters for HIGH_T 19
- Disregarded resistance measurements: 21
- Considerations on the origin of 'O third' in HIGH_T 22

References 24

Experimental Section

Sample sensor preparation

The sensor substrates, prepared by laser-scribing an alumina tile (0.38 mm thickness, 99.6%, Laser Cutting Ceramics Ltd) into 8x8 mm squares, were coated with 1 µm of Au (99.99%, Kurt J. Lesker) by e-beam evaporation (at 30 °C and 2.5×10^{-6} mbar base pressure, deposition rate 2.6 Å s^{-1}). Subsequently, the electrodes were etched via photolithography, using an S1818 positive photoresist and light exposure of 130 mJ cm^{-2}, followed by etching for 3600 s, using MF319 as a developer. The interdigitated electrodes produced in this way, as shown in Figure S1, had a separation between the digits of 25 µm.

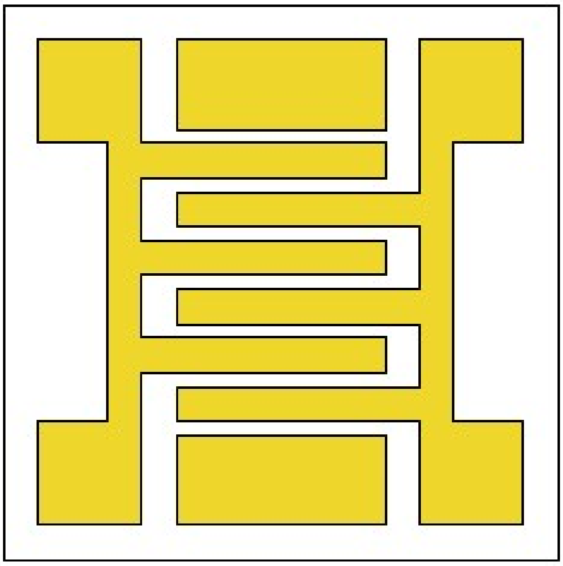

Figure S1: Layout of the electrodes on an alumina sensor platform. The electrodes are drawn to scale, with the sides of the substrate being 8 mm and the separation between the electrodes' digits being 25 µm (in the vertical direction on this diagram). The insulated Au pads were added as contact pads for the thermocouple to facilitate heat distribution and ensure accurate temperature measurements.

The sensitive layer was prepared by drop-casting an SnO_2 suspension onto the sensor substrates. The suspension was prepared from SnO_2 powder (Alfa Aesar, 99.995% purity) ground with EtOH (Fisher, absolute purity) and Polyethylene Glycol 600 (Sigma Aldrich). Subsequently, the sensors were calcined in 30 ml min^{-1} O_2 flow (N5.5 purity, BOC) at 800 °C for 5 days, cooled under the same atmosphere to below 50 °C and sealed hermetically until insertion into the load lock of the spectrometer.

Instruments used

The LOW_T and HIGH_T experiments were performed at the B07−C beamline of the Diamond Light Source synchrotron[1]. The synchrotron radiation energy was set to 895 eV and 2000 eV and calibrated using the Au 4f peak; the position of the Au 4f emission was measured after every monochromator change, and the binding energy scale of the Au and SnO_2 spectra collected after that change was adjusted so that Au $4f_{7/2}$ = 84.0 eV.

The beamline is equipped with a Phoibos 150 NAP hemispherical 2D analyser, operating in the magic-angle configuration. High-resolution Sn 3d and O 1s scans were collected at 15 eV pass energy and a step of 0.05 eV for the 895 eV photon energy spectra and 30 eV pass energy with 0.1 eV step for the 2000 eV photon energy spectra. The C 1s spectra were collected at 30 eV pass energy for both the 895 eV and 2000 eV spectra due to its low signal-to-noise ratio at 15 eV. Because of this, the intensity of the C 1s spectra is not directly comparable to Sn 3d and O 1s spectra, as the larger pass energy increases the recorded counts per second and thus the absolute intensity of the spectra.

N6.0 O_2 and N5.5 H_2 gases were fed to the analysis chamber via separate piezoelectric leak valves, and pressure was maintained constant via a butterfly valve. The analysis chamber and the gas lines feeding it were baked out immediately before the beamtime to limit possible contaminants. The sample was heated by a resistive heater mounted within the sample stage assembly, and the temperature was monitored by a K-type (chromel–alumel) thermocouple mounted on the sample stage and pressed directly against the sensor plate onto an Au pad electrically insulated from the sensor electrodes. Resistance measurements were collected by grounding the sensor to the system's sample manipulator and using the 'Bias' lead of the sample stage as the positive terminal. A constant potential of 100 mV was applied, and the resulting current was measured by a Stanford SR570 source meter.

Data acquisition procedure

To ensure accurate calibration for each series of spectra the gold of the sensor electrode was used as a reference. After collecting the Au 4f calibration spectra on the grounded sensor electrode, the sample was returned to the same analysis spot (ca. 200 µm in diameter) on the SnO_2 sample, with the motorised stage providing control to ± 10 µm in the (XY) plane parallel to the sample's surface (i.e. much smaller than the analysis spot size, with adjustments in the sample – analyser (Z) axis Z-direction optimised in 5 µm increments in order to maximise signal intensity (typically a plateau in intensity was observed over a range ± 10 µm in the Z-direction). The stage position was then left unchanged until all scans of the current step were collected, and subsequently moved only for the following Au binding energy calibration.

In order to ensure comparable intensities of the XPS spectra, and hence provide reliable determination of the O/Sn ratio and in turn oxygen vacancy concentration, the experiments were carried out at constant temperature to eliminate thermal movement of the sample stage (which could affect the observed intensities of the spectral peaks over time) by allowing the system sufficient time to stabilise (at least 2 h after reaching stable temperature before measurements start).

In order to further limit uncertainty over temporal differences in peak intensity arising during spetra collection, the Sn 3d, O 1s and C 1s regions were collected sequentially, with this three-region cycle repeated multiple times before peak averaging the obtained data. Therefore, the effects of any possible transient changes in peak intensity were evenly spread out over all regions, affecting all intensities equally (no intrinsic bias in results for any one spectral peak). In UHV, three scans were collected in each region collected at 895 eV and two scans for the 2000 eV spectra. During O_2 exposure, the number of scans was doubled to mitigate the decreased signal-to-noise ratio under NAP conditions.

The intensity of the incident synchrotron beam was monitored throughout the experiments to ensure that the peaks area are not affected by the changes in excitation radiation. Moreover, the Diamond Light Source synchrotron employs a ring 'top-up' mode in order to maintain a constant beam intensity. Therefore, the spectra discussed are not affected by the changes in beam intensity.

Given the precautions taken, no significant relative change is expected in the data collected; this is confirmed by the lack of change in the intensity between subsequent single scans collected for each region. For example, the consequent scans of the Sn 3d and O 1s region collected during step E1 of experiment LOW_T is presented in Figure S2. Each scan was processed individually, and the derived quantification parameters are presented in Table S1. In all instances, the relative standard deviation is below 1%, indicating no significant transient changes between the spectra collected during each step. Considering that the spectral noise affects the O 1s peak fit, the area is reported as 'O total', which is the sum of 'O lattice' and 'O third', and the O/Sn peak ratio in this instance is calculated using the raw values of Sn $3d_{5/2}$, Sn $3d_{3/2}$ and 'O total'.

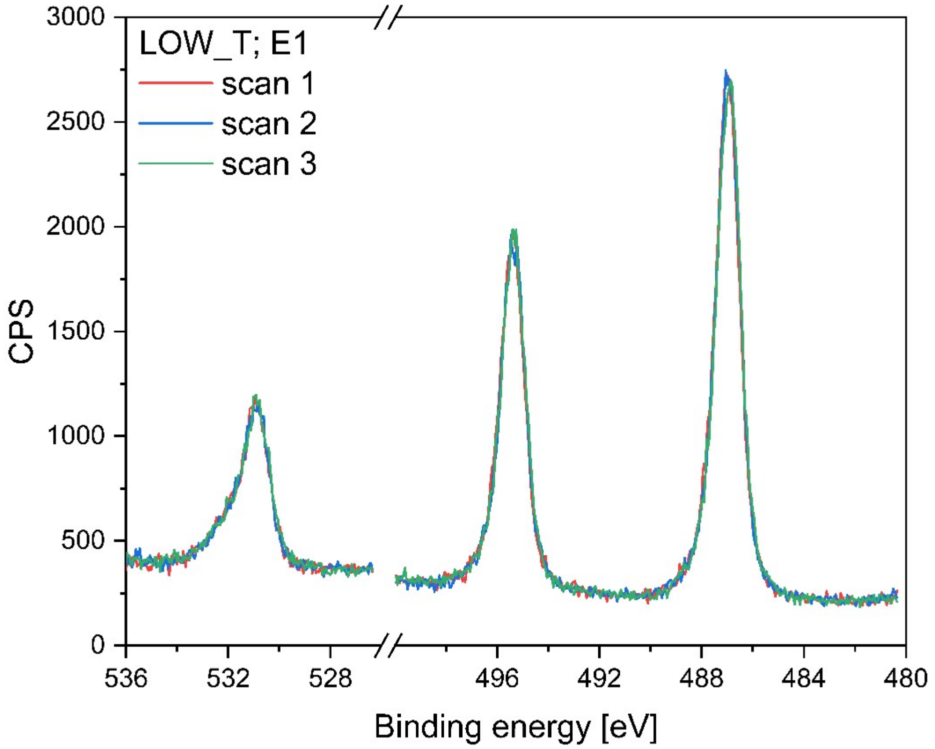

Figure S2: Overlay of the Sn 3d and O 1s single scans collected during step E1 of experiment LOW_T, showing that there is no thermal drift of the sample that would cause changes in the spectra intensity.

Table S1: Quantification parameters derived from the Sn 3d and O 1s spectra presented in Figure S2. Raw peak areas of the Sn 3d and O 1s ('O total') were used to calculate the O/Sn peak ratio to show that the relative spectral intensities are not changing during the course of a step.

	Sn $3d_{5/2}$ p.pos [eV]	Sn $3d_{5/2}$ FWHM [eV]	Sn $3d_{5/2}$ area	Sn $3d_{3/2}$ area	'O total' area	O/Sn peak ratio
scan 1	486.95	1.09	3329	2251	1308	0.234
scan 2	486.92	1.09	3365	2262	1289	0.229
scan 3	486.90	1.10	3364	2271	1306	0.232
Average	486.92	1.09	3352	2261	1301	0.232
STD	0.02	0.01	16.85	8.348	8.357	0.002
Rel. STD	0.004	0.431	0.503	0.369	0.642	0.917

Signal attenuation by gas under NAP conditions

Another factor affecting the estimated O/Sn ratio is the attenuation of photoelectrons by gas molecules under NAP conditions. This affects lower kinetic energy photoelectrons more than higher energy ones, which would result in an underestimation of the O/Sn ratio during gas exposure. This was accounted for by preparing isobaric calibration curves of intensity as a function of the kinetic energy of photoelectrons.

The Au 4f region scans were collected at different O_2 pressures and photon excitation energies s to produce a set of spectra whose intensity changed as a function of both pressure and photoelectron kinetic energy. The spectra were fitted with Doniach-Sunjic (DS) components, and the areas of peaks collected under increasing oxygen pressure at each kinetic energy were expressed as a fraction of the UHV peak's area. Subsequently, the fractions for each pressure were fitted with a logarithm function (general formula y=logA+B) to produce a curve for estimation of attenuation of photoelectrons at a given pressure as a function of the kinetic energy of electrons, as shown in Figure S3. Therefore, the quantification of peaks under gas ambient can be compared as if the spectra were collected in UHV, where their separation on the BE scale does not affect quantification results. The errors associated with this calibration were estimated by calculating the 95% confidence interval of the Au 4f gas/UHV ratio at relevant kinetic energies, and are presented in Table S3.

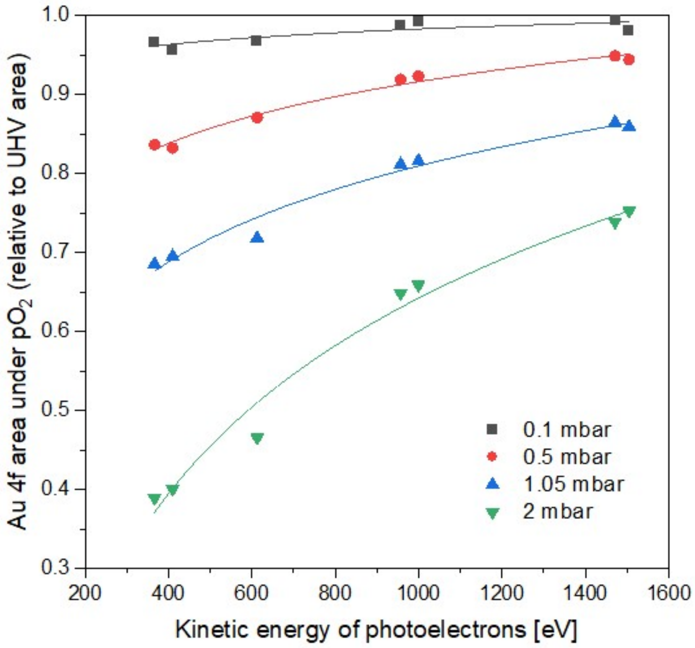

Figure S3: Attenuation calibration – curve fitting for the energy-dependent attenuation expressed as a ratio of the Au 4f peaks collected under the relevant pressure (0.1, 0.5, 1.05 or 2.0 mbar) and in UHV.

Sampling depth estimation

The sampling depth of the 'shallow' and 'deep' spectra was estimated using two methods. The first method involved digitising (using OriginPro) a plot of the electron inelastic mean free path (IMFP) as a function of the kinetic energy of photoelectrons.[2] Based on the obtained data points, an exponential regression was applied to allow the estimation of energy-dependent analysis depth for O 1s and Sn 3d peaks. The fitting is presented in Figure S4. Analysis depth in XPS is normally cited as three times the IMFP, which indicates the depth below which 95% of the signal is attenuated. The analysis depths determined using this method are presented in Table S2.

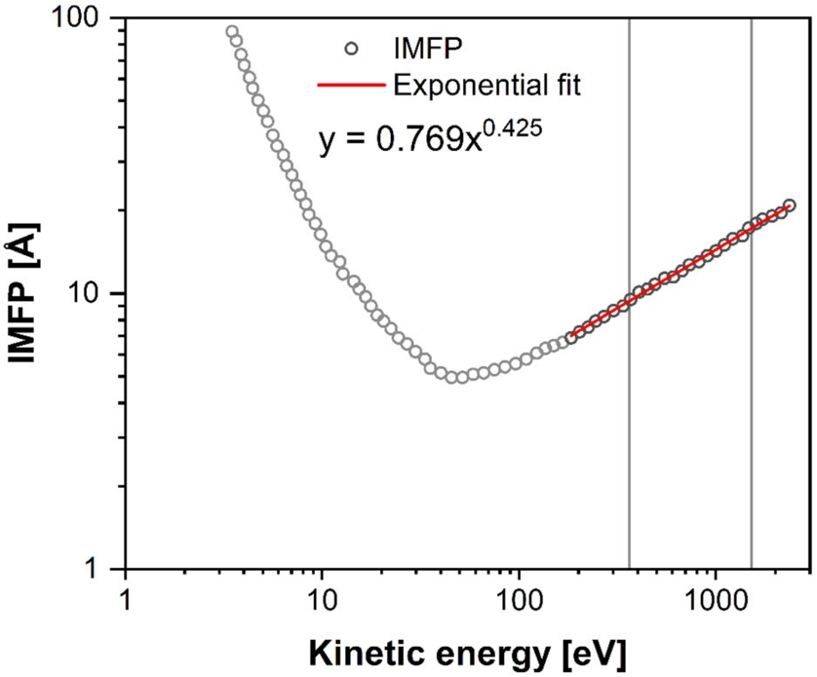

Figure S4: Plot of energy-dependent inelastic mean free path (IMFP) of photoelectrons for inorganic solids based on digitised plot obtained from reference [2]. Grey lines indicate the energy range over which IMFP information was extracted.

Table S2: MPF and analysis depth of O 1s and Sn 3d regions for 'shallow' and 'deep' spectra as estimated from the fitted digitised data presented in Figure S4.

	Kinetic energy [eV]	IMFP [nm]	Analysis depth [nm]	Average analysis depth [nm]
O 1s (895 eV)	365	0.942	2.82	2.89
Sn 3d (895 eV)	407	0.986	2.96	
O 1s (2000 eV)	1470	1.70	5.10	5.13
Sn 3d (2000 eV)	1512	1.72	5.16	

In order to examine the accuracy of the estimations presented above, the analysis depth was also estimated using the Tanuma Powell and Penn algorithm,[3] as employed in the QUASES-IMFP-TPP2M software. Input parameters were as follows: density 6.95 g cm^{-3}, atomic mass 150.71, band gap energy 3.6 eV and 16 valence electrons per unit formula. Based on these parameters, the estimated IMFP and analysis depths are presented in Table S3.

Table S3: MPF and analysis depth of O 1s and Sn 3d regions for 'shallow' and 'deep' spectra as estimated from the IMFP-TPP2M algorithm using QUASES software.

	Kinetic energy [eV]	IMFP [nm]	Analysis depth [nm]	Average analysis depth [nm]
O 1s (895 eV)	365	0.887	2.66	2.76
Sn 3d (895 eV)	407	0.952	2.87	
O 1s (2000 eV)	1470	2.41	7.23	7.32
Sn 3d (2000 eV)	1512	2.47	7.41	

Given that both these sets of values are rough estimates, the analysis depth for 'shallow' and 'deep' spectra is not quoted to the same number of significant figures but approximated as 2.5 nm for the former and 5 nm for the latter.

Spectra analysis

The spectra were processed in CasaXPS software. The binding energy (BE) scale of spectra collected using synchrotron radiation was calibrated by fitting the Au 4f peak with two Doniach-Sunjic (DS) components and adjusting the BE scale so that the Au $4f_{7/2}$ peak appears at 84.0 eV. Background subtraction was performed using the Shirley method for the Au 4f region, as well as the O 1s and C 1s regions used for sample characterisation. Tougaard background was used for the Sn 3d peak, which was found to be more appropriate than Shirley in this case, as the Shirley background appeared above the measured intensity between the Sn $3d_{5/2}$ and Sn $3d_{3/2}$ peaks.

The Sn 3d peaks were fitted with two Lorentzian Asymmetric lineshape (LF) components, one for each peak of the 3d doublet. The lineshape parameters of the two components were adjusted to fit the spectra taken immediately before the first oxygen exposure ('UHV before') and remained unchanged within each experiment. No constraints were imposed on the peak position, area or full width at half maximum (FWHM).

The O 1s peak was fitted with two components, the lattice peak, which corresponds to the oxygen contained within the SnO_2 lattice, and 'O third', corresponding to all other oxygen present within the analysed volume. The shape of the lattice component was determined by an LF lineshape whose parameters were adjusted to match the experimental data during the first step of investigation (step 'UHV before'), i.e., after reduction and dehydroxylation but before oxygen exposure, when the sample is notionally free of adsorbates and hydroxyls. Any changes to the shape of subsequent photoemission measurements are modelled by the 'O third' component, which uses a symmetrical Lorentzian lineshape (LA). Since the lattice peak in 'UHV before' is fitted to match the O 1s photoemission, the resulting area of 'O third' is zero or negligible. Therefore, the lineshape parameters of 'O third' are adjusted to achieve the best fit to spectra after the first introduction of oxygen (D1). The components were fitted to data points using the Marquardt algorithm for least-squares curve fitting. Uncertainty in the O 1s region quantification was estimated by averaging the FWHM of lattice components during an experiment and creating three new spectra fits, one where the FWHM of lattice peak is constrained to the average value, and two for upper and lower boundaries of the uncertainty in BE measurement (FWHM compound uncertainty of ±0.04 eV for 895 eV and ±0.07 eV for 2000 eV spectra). All other parameters remained unconstrained, including those of the 'O third' component.

The C 1s region was fitted with four Gaussian-Lorentzian (GL) components corresponding to hydrocarbons (C-H), alcohols (C-OH), ketones (C=O) and esters (O-C=O). The FWHM of C-OH, C=O and O-C=O were constrained to be the same as the FWHM of the C-H peak, and their BE position was constrained to, respectively, 1.5, 2.5 and 4.0 eV above the C-H peak. The C 1s region and components within were not used in atomic composition calculations due to the lack of accurate relative sensitivity factors (RSF), which are only tabulated for some excitation energies, for example, the Al kα.

Table S4: Errors associated with the intensity calibration under NAP conditions calculated as half the difference between the 95% confidence interval values of the fitting of data points shown in Figure S3.

	Intensity calibration error for Sn 3d and O 1s photoemissions [%]			
	'shallow' spectra (895 eV)		'deep' spectra (2000 eV)	
pressure of O_2	Sn 3d (KE = 408 eV)	O 1s (KE = 364 eV)	Sn 3d (KE = 1513 eV)	O 1s (KE = 1469 eV)
1 mbar	3.07	3.48	2.67	2.89
2 mbar	9.42	11.3	5.30	5.63

The O/Sn and 'O third'/Sn ratios were calculated by dividing the areas of the O components by the sum of the two components of the Sn 3d emission normalised using RSF values (2.93 for O 1s and 25.1 for Sn 3d). Although the RSF values were derived for Al kα radiation, they may be used for other energies if relative, rather than absolute, abundance is considered, as in the case of this experiment. Additionally, the ratios calculated from spectra collected in non-UHV conditions were adjusted by the ratio determined from attenuation calibration. The uncertainty associated with these ratios was calculated as the square root of the sum of squared uncertainties in component ratio and attenuation calibration.

LOW_T

In situ reduction

Prior to the experiment, the sensor was reduced in situ by heating in hydrogen. The process was monitored with both the resistance measurements and 'shallow' XPS analysis, as shown in Figure S5.

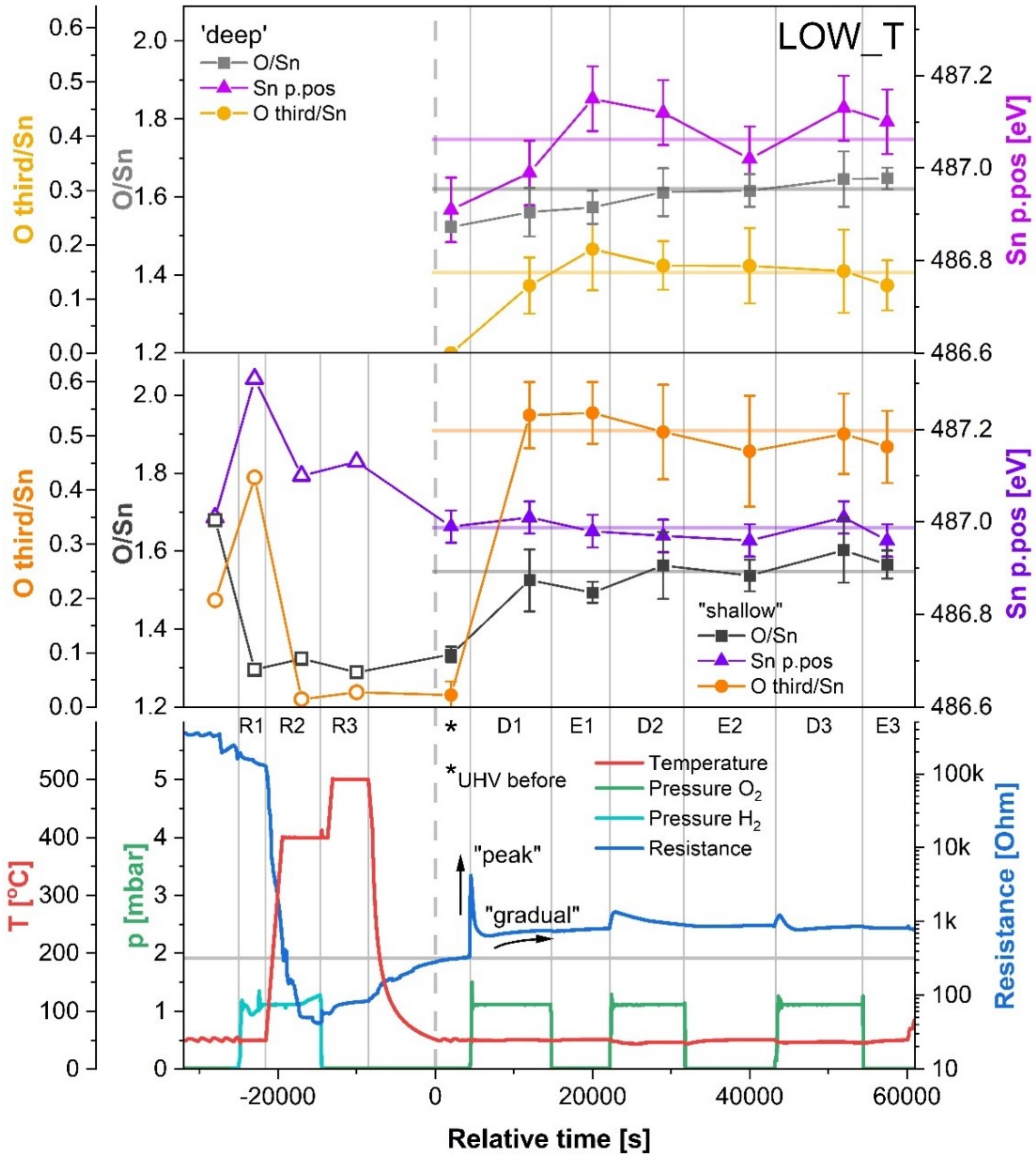

Figure S5: Extended version of the LOW_T results, including the resistance and XPS measurements collected during the in situ reduction. Hollow points indicate data collected less rigorously, in different atmospheres and at different temperatures, which may affect the quality of the data; hence, no error bars are presented for these points. Sn p.pos denotes the binding energy position of the Sn $3d_{5/2}$ peak.

It should be noted that the spectra collected during the reduction procedure were intended to give a broad picture of the changes occurring at the surface, and the data acquisition procedure was less rigorous than during the actual experiment, as described in the main paper (for example, the sudden

large changes in temperature could cause thermal drift of the sample stage over the period of data collection). Therefore, error bars for the corresponding data points are not presented, and conclusions should be drawn from the data carefully.

The reduction began by exposing the sensor to 1 mbar H_2 at 50 °C (R1 in Figure S5). This resulted in a gradually progressing decrease in resistance, from the initial value of 300 kΩ (prior to any treatment) to about 100 kΩ towards the end of R1. During this time, the area of the O 1s lattice component (O/Sn in Figure S5) decreased from 1.70 to 1.30, and the area of the 'O third' component ('O third'/Sn) increased from 0.20 to over 0.40. These changes are consistent with the reaction of H_2 with surface lattice oxygen atoms to transform them into surface hydroxyls.[4] Furthermore, upward band bending of approximately 0.30 eV was observed (Sn p.pos increased from 487.00 to 487.30 eV), which is also consistent with the formation of electron donors, for example, rooted hydroxyls (hydroxyl groups where the oxygen atom is placed on a bridging oxygen surface site, i.e. a bridging surface oxygen atom with a hydrogen atom attached to it).[4]

After collecting the initial spectra shortly after first inletting H_2, the temperature was increased to 400 °C for 1 h (R2 in Figure S5), which resulted in a pronounced decrease in resistance. While this is consistent with normal semiconductor behaviour, where more donors can be ionised as the temperature increases, the resistance continued to decrease even after the temperature stabilised at 400 °C, from 100 kΩ to circa 50 Ω, the lowest value observed in this experiment. Accompanying this is the almost complete removal of surface hydroxyls ('O third'/Sn decreases to close to 0), which can be explained if the hydroxyls donors were replaced by oxygen vacancy donors; without surface oxygen atoms, no more hydroxyls can be created even in the presence of H_2. However, the O/Sn ratio does not decrease further, which could indicate that only the surface oxygen atoms converted into hydroxyls in R1 become vacancies in R2 or that nearly all surface oxygen atoms were converted into hydroxyls. An additional decrease in resistance on top of that caused by oxygen vacancies could come from interstitial hydrogen atoms incorporated into the lattice during H_2 treatment.[5] Whereas the resistance reached its minimum value in this step, the position of the Sn 3d peak changed to 487.10 eV, a decrease of 0.20 eV compared to the previous step. While this should indicate upward band bending (and decreased resistance), possibly caused by the removal of surface hydroxyls, it is not entirely consistent with the low resistance observed.

During R3, the H_2 was evacuated to UHV, and the temperature was increased to 500 °C for 1 h to dehydroxylate the surface and allow H interstitials to diffuse out of the surface. A decrease in donors density is inferred from resistance measurements, which show a slight increase, from 50 Ω to 80 Ω, despite the increasing temperature that typically lowers the semiconductor's resistance. However, the 'O third' component was already removed in R2, and the O/Sn ratio does not appear to decrease any further. Band bending also appears to remain at the same level as in R2.

Finally, the heater was turned off, and the sample was passively cooled to the experimental temperature of 50 °C, thus ending the in situ reduction procedure. This resulted in an increase in resistance up to about 300 Ω, which is taken as the final value of resistance after the reduction and the starting point of the experiment LOW_T. This was not accompanied by any changes in either O/Sn or 'O third'/Sn. However, the Sn 3d peak shifted back to the original value of 487.00 eV, which is not easily explained given the overall change in the O/Sn stoichiometry from before R1. It is possible that at such low temperatures, the oxygen vacancies are not sufficiently ionised to cause band bending. However, the decreased sensor resistance points to a considerably increased conductivity, which implies vacancy ionisation and donation of electrons into the conduction band.

Sn 3d and O 1s fit parameters for LOW_T

Table 5: Fit parameters of the 895 eV 'shallow' spectra in the LOW_T experiment.

Step	Parameter	Sn 3d 5/2	Sn 3d 3/2	O lattice	O third
UHV before	BE [eV]	486.93	495.35	530.78	N/A
	FWHM	1.18	1.18	1.14	N/A
	Area	4539.53	3022.42	1206.49	0.00
	Lineshape	LF	LF	LF	LA
D1	BE [eV]	486.97	495.39	530.83	532.11
	FWHM	1.09	1.08	1.09	1.72
	Area	2302.42	1579.20	674.01	228.31
	Lineshape	LF	LF	LF	LA
E1	BE [eV]	486.93	495.35	530.78	531.99
	FWHM	1.10	1.11	1.15	1.72
	Area	3356.56	2273.58	995.51	283.34
	Lineshape	LF	LF	LF	LA
D2	BE [eV]	486.92	495.34	530.78	532.08
	FWHM	1.08	1.08	1.12	1.30
	Area	2376.44	1618.83	731.61	167.56
	Lineshape	LF	LF	LF	LA
E2	BE [eV]	486.89	495.31	530.74	531.99
	FWHM	1.11	1.11	1.13	1.55
	Area	3374.51	2301.07	1008.27	240.21
	Lineshape	LF	LF	LF	LA
D3	BE [eV]	486.95	495.38	530.81	532.13
	FWHM	1.08	1.08	1.08	1.39
	Area	2273.39	1531.73	700.70	178.45
	Lineshape	LF	LF	LF	LA
E3	BE [eV]	486.89	495.31	530.73	532.04
	FWHM	1.11	1.10	1.12	1.47
	Area	3187.38	2149.71	975.10	260.24
	Lineshape	LF	LF	LF	LA

Table 6: Fit parameters of the 2000 eV 'deep' spectra in the LOW_T experiment.

Step	Parameter	Sn 3d 5/2	Sn 3d 3/2	O lattice	O third
UHV before	BE [eV]	486.91	495.33	530.80	N/A
	FWHM	1.40	1.40	1.35	N/A
	Area	6666.36	4634.25	2007.51	0.00
	Lineshape	LF	LF	LF	LA
D1	BE [eV]	486.99	495.41	530.90	532.22
	FWHM	1.40	1.39	1.37	2.25
	Area	8864.73	6185.89	2788.83	168.01
	Lineshape	LF	LF	LF	LA

E1	BE [eV]	487.15	495.57	531.05	532.29
	FWHM	1.40	1.37	1.36	1.82
	Area	8879.27	6110.84	2804.37	198.68
	Lineshape	LF	LF	LF	LA
D2	BE [eV]	487.12	495.54	531.02	532.43
	FWHM	1.38	1.37	1.32	2.49
	Area	7062.16	4880.77	2175.68	279.59
	Lineshape	LF	LF	LF	LA
E2	BE [eV]	487.02	495.44	530.91	532.21
	FWHM	1.40	1.37	1.29	2.18
	Area	8057.34	5488.62	2441.45	332.62
	Lineshape	LF	LF	LF	LA
D3	BE [eV]	487.13	495.54	531.01	532.29
	FWHM	1.36	1.34	1.33	2.41
	Area	6716.89	4655.00	2128.46	274.47
	Lineshape	LF	LF	LF	LA
E3	BE [eV]	487.10	495.52	530.95	532.24
	FWHM	1.35	1.35	1.28	2.21
	Area	8130.66	5715.74	2498.88	377.65
	Lineshape	LF	LF	LF	LA

Considerations on the origin of 'O third' in LOW_T during O_2 dosing

Firstly, the possibility of 'O third' being surface hydroxyls is considered. Figure S6 shows the O 1s spectrum recorded during exposure of the surface to 1 mbar H_2 at 50 °C as part of the in situ reduction (R1 in Figure S5), which resulted in the appearance of a peak that can undoubtedly be assigned to surface hydroxyls and is similar to 'O third' observed during O_2 dosing (Figure 3, main article). The onset of this peak is also visible in the 'O third' trace in step R1 in Figure S5, as well as its subsequent disappearance during surface dehydroxylation (R2 in Figure S1, middle panel), indicating the process was successful. In order to minimise interference of surface hydroxyls during the experiment, high purity oxygen (N6.0, 99.9999%) was used, with a moisture content of below 0.5 ppm and H_2 content below 0.05 ppm. Because of this, the sensor would have to be exposed to the high purity O_2 gas for over a million hours to receive the same absolute amount of hydrogen dosed during one hour of H_2 exposure (at the same partial pressure). Instead, within less than three hours of O_2 introduction, which is the duration of the D1 step, the 'O third' signal surpasses that obtained as a result of H_2 exposure in R1 (compare 'O third'/Sn between R1 and D1 in Figure S5, middle panel). Whilst we have no direct evidence that can rule out the formation of surface hydroxyls, the adsorption energies of O_2 and H_2O, which compete for the same adsorption sites, are comparable,[6–8] and given the six orders of magnitude larger O_2 partial pressure relative to the maximum possible H_2O partial pressure suggest that O_2 should occupy the available adsorption sites quickly, thus preventing surface hydroxylation due to dissociative H_2O adsorption. Therefore, it is unreasonable to assign the 'O third' peak resulting from O_2 exposure to only surface hydroxyls, even though they may still contribute an inconsequential fraction of the observed intensity of the 'O third' peak.

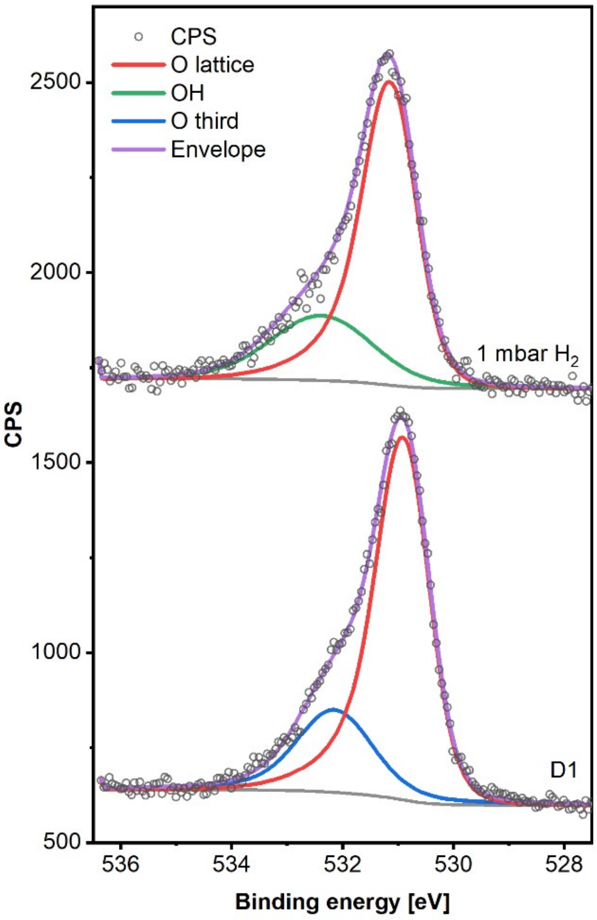

Figure S6: O 1s high-resolution spectra collected at 50 °C in 1 mbar H2 as part of the in situ reduction procedure and 1 mbar O2 during the D1 step of the LOW_T experiment. Spectra were normalised with respect to their corresponding Sn 3d peaks.

Another possible source of 'O third' photoemission is oxygen atoms in organic molecules constituting adventitious carbon contamination, which is ever-present in virtually all UHV systems. Although some carbonaceous species were deposited onto the sensor's surface during in situ reduction and remained throughout the experiment, these also cannot account for the observed 'O third' emission. As observed in 'shallow' spectra, the photoemission from carbonaceous species is largest at the start of the experiment (UHV before) and decreases slowly throughout the experiment, as shown in Figure S7. The C 1s spectra were fitted with three 'oxidised' components to estimate the amount of oxygen bound in the contaminants (details in the Experimental section). Relative to the 'UHV before' step (during which the area is 1 by definition – at the start of the experiment), the estimated amount of organic oxygen decreases gradually after every oxygen exposure and reaches 0.2 during step E3, indicating combustion of carbonaceous species, with the products leaving the surface. This hypothesis is supported by the change in the relative abundance of 'oxidised' carbon peaks; as the experiment progresses, the high oxidation state 'O-C=O' peak becomes dominant while the 'C-O' peak diminishes. Therefore, if organic oxygen gave rise to the observed 'O third' intensity, one would expect a similar fivefold decrease in 'O third' during the experiment (note, as mentioned previously, the different pass energies used for collection of C 1s

and O 1s spectra prevent a direct correlation of peak areas). Instead, the ‘O third’/Sn ratio is around zero during ‘UHV before‘, when oxidised C 1s intensity is at its maximum, and then increases to 0.5 and remains constant despite organic oxygen disappearing from the surface. Therefore, the contribution of the organic oxygen to the observed O 1s photoemission must be small enough to be accounted for in the slight asymmetry of the lattice peak (as its parameters were fitted to match the emission during UHV before), and it therefore does not contribute to the ‘O third’ emission.

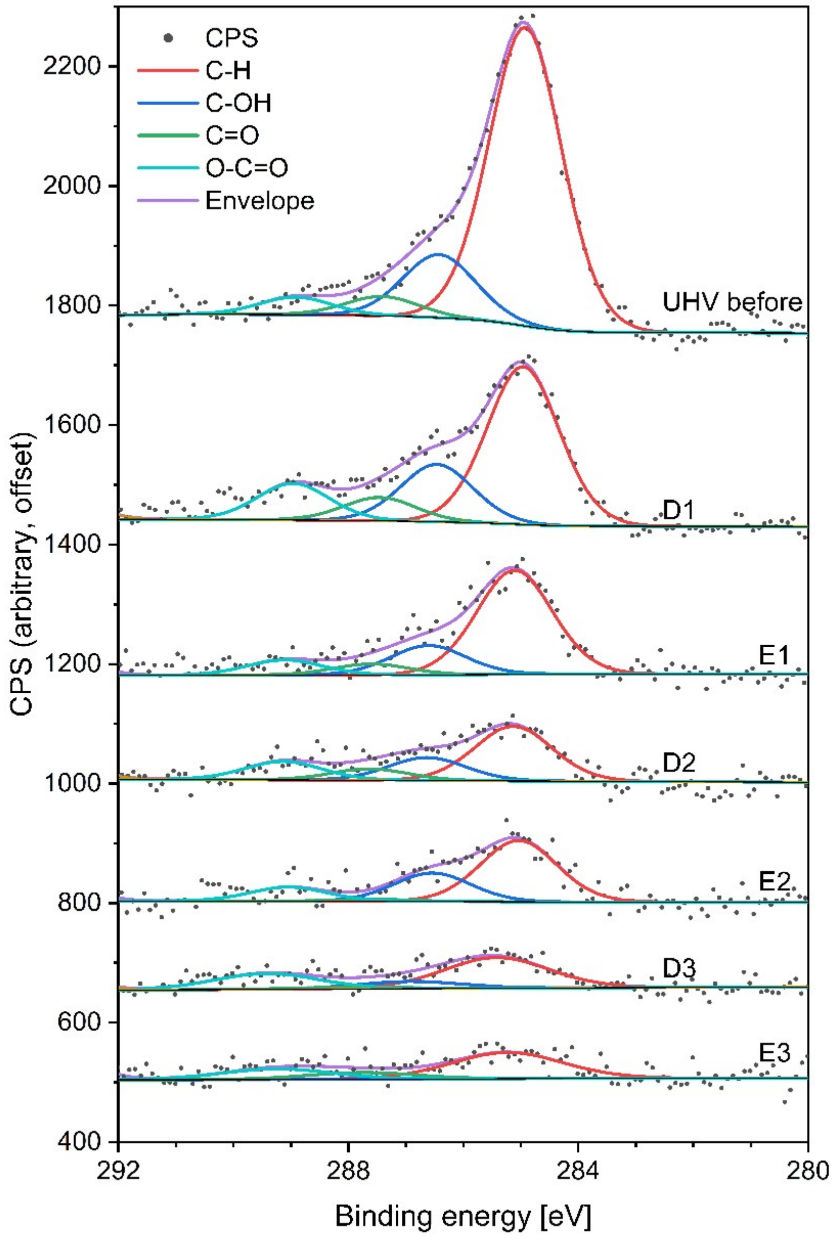

Figure S7: Comparison of the C 1s emission (collected with a 30 eV pass energy) during subsequent UHV steps of the low-temperature experiment. The amount of organic contamination on the surface decreases steadily throughout the experiment. CPS was normalised with respect to the corresponding Sn 3d peaks to ensure intensity comparability.

Magnification of D1 resistance change

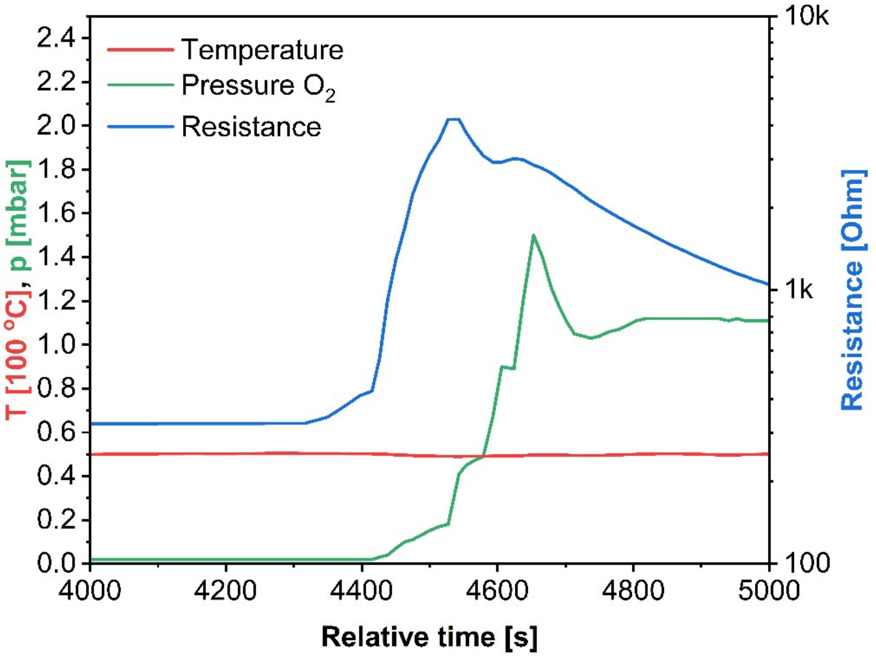

Figure S8: Magnification of the resistance, temperature and pressure measurements at the start of D1 step of experiment LOW_T. The resistance decrease begins before the maximum pressure of O_2 is reached.

HIGH_T

In situ reduction

Prior to the experiment, the sensor was reduced in situ by heating in hydrogen. The process was monitored with both the resistance measurements and 'shallow' XPS analysis, as shown in Figure S9.

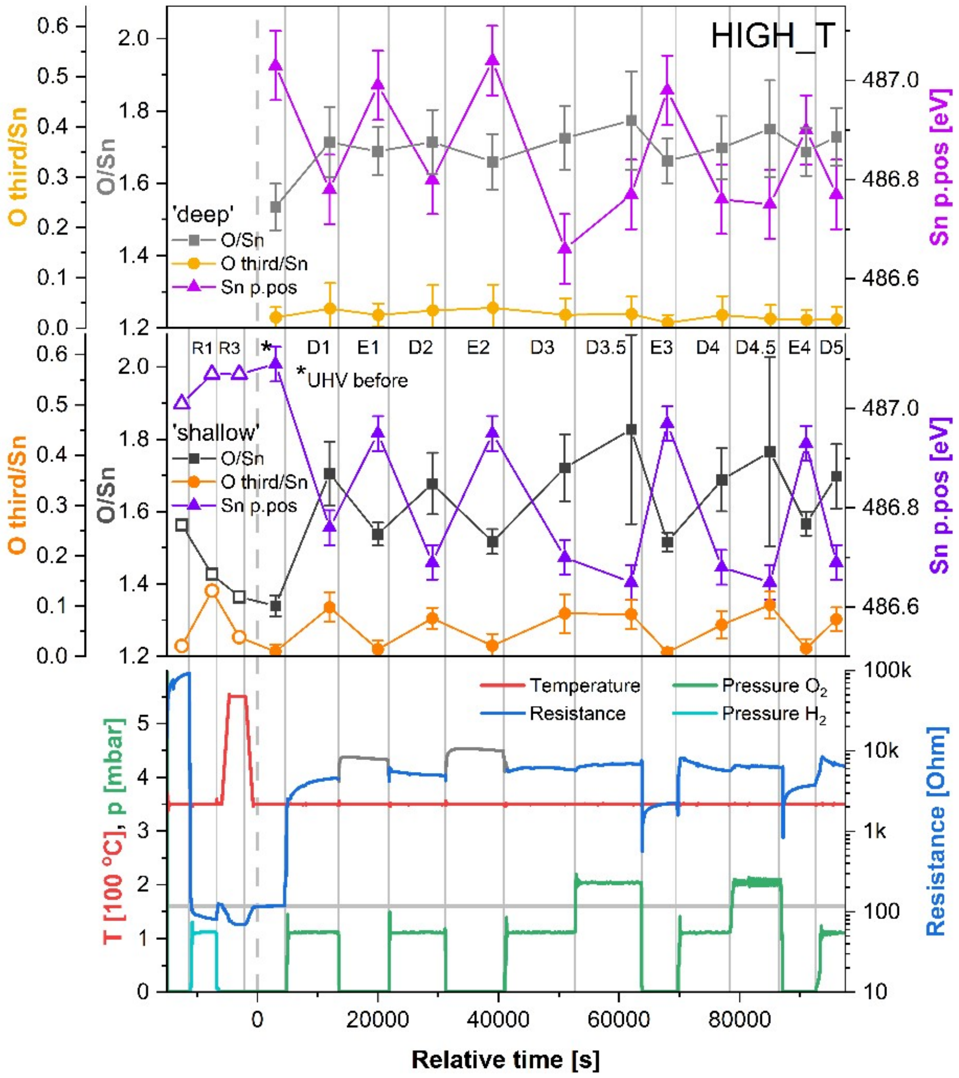

Figure S9: Extended version of the HIGH_T results, including the resistance and XPS measurements collected during the in situ reduction. Hollow points indicate data collected less rigorously, in different atmospheres and at different temperatures, which may affect the quality of the data; hence, no error bars are presented for these points. Sn p.pos denotes the binding energy position of the Sn 3d$_{5/2}$ peak.

The reduction began by exposing the sensor to 1 mbar H_2 at 350 °C, indicated in Figure S9 as R1. This resulted in a decrease in the O/Sn ratio by about 0.15, from just under 1.60 to just over 1.40. At the same time, the 'O third' emission emerged, reaching the 'O third'/Sn ratio of 0.15. Both these changes are consistent with the transformation of surface oxygen atoms into rooted hydroxyls, which act as electron donors. Additionally, the resistance decreased substantially, from the initial value of 100 kΩ prior to any treatment down to 80 Ω at the end of R1, also consistent with the formation of oxygen vacancies or rooted hydroxyls. While a marked decrease in resistance is

observed, the bands bend downwards relatively by a little, with the Sn p.pos increasing from 487.00 to 487.10 eV.

Since the data acquisition of the R1 spectra took approximately 1 hour, during which the sensor was already exposed to high-temperature reducing conditions, the next step of increasing the temperature to 400 °C (R2 in LOW_T experiment) was omitted. Instead, the H_2 was evacuated to UHV, and the sensor's temperature was increased to 550 °C for dehydroxylation, denoted as R3 in Figure S9.

During R3, the resistance started to increase up to 125 Ω initially upon the evacuation of H_2 but then started to decrease again when the temperature was increased to 550 °C, eventually stabilising at about 70 Ω. At the same time, the surface hydroxyls were removed, as indicated by the disappearance of the 'O third' component (R3, Figure S9, middle panel). The O/Sn ratio decreased by just under 0.10 compared to R1, down to about 1.35. Both the disappearance of 'O third' and the decrease in O/Sn indicate the formation of surface vacancies via the desorption of rooted hydroxyls and surface oxygen atoms, respectively. The Sn p.pos remained unchanged, still increased compared to before H_2 introduction in R1, indicating that the bands remain bent downwards. Since one type of electron donors (rooted hydroxyls) was largely replaced with another donor (oxygen vacancies), the insignificant change in band bending during R3 is unsurprising.

Following R3, the temperature of the sensor was decreased back to 350 °C, which did not significantly affect either of the O/Sn ratio, the 'O third' component or the Sn p.pos, with their values relative to those from before R1, indicating surface reduction occurred during the reduction procedure. As a result of the cooling of the sensor, the resistance increased slightly, from 70 Ω to 120 Ω, which was the final resistance value after the reduction and the starting point for the following HIGH_T experiment.

Sn 3d and O 1s fit parameters for HIGH_T

Table 7: Fit parameters of the 895 eV 'shallow' spectra in the HIGH_T experiment.

Step	Parameter	Sn 3d 5/2	Sn 3d 3/2	O lattice	O third
UHV before	BE [eV]	487.04	495.46	530.82	532.70
	FWHM	1.29	1.27	1.25	1.00
	Area	2349.41	1546.99	644.12	0.10
	Lineshape	LF	LF	LF	LA
D1	BE [eV]	486.72	495.14	530.47	532.13
	FWHM	1.21	1.17	1.32	1.07
	Area	1219.59	807.60	409.11	23.30
	Lineshape	LF	LF	LF	LA
E1	BE [eV]	486.90	495.33	530.65	532.00
	FWHM	1.26	1.25	1.34	0.94
	Area	2001.26	1334.42	624.80	7.20
	Lineshape	LF	LF	LF	LA
D2	BE [eV]	486.64	495.06	530.37	531.94
	FWHM	1.22	1.20	1.35	0.90
	Area	1174.64	782.40	390.67	16.06
	Lineshape	LF	LF	LF	LA
E2	BE [eV]	486.90	495.33	530.65	532.11

	FWHM	1.26	1.25	1.34	0.90
	Area	2150.03	1438.78	670.69	7.44
	Lineshape	LF	LF	LF	LA
D3	BE [eV]	486.65	495.07	530.39	532.10
	FWHM	1.19	1.20	1.32	1.27
	Area	1386.22	942.12	474.89	21.09
	Lineshape	LF	LF	LF	LA
D3.5	BE [eV]	486.61	495.04	530.36	532.20
	FWHM	1.19	1.17	1.32	1.06
	Area	1111.31	757.99	383.50	18.58
	Lineshape	LF	LF	LF	LA
E3	BE [eV]	486.92	495.34	530.68	532.34
	FWHM	1.25	1.24	1.32	0.90
	Area	2312.09	1548.97	710.57	3.02
	Lineshape	LF	LF	LF	LA
D4	BE [eV]	486.64	495.06	530.36	532.08
	FWHM	1.20	1.19	1.34	0.90
	Area	1414.42	958.24	477.29	13.07
	Lineshape	LF	LF	LF	LA
D4.5	BE [eV]	486.60	495.03	530.33	532.13
	FWHM	1.20	1.19	1.31	1.04
	Area	930.30	633.76	314.58	17.36
	Lineshape	LF	LF	LF	LA
E4	BE [eV]	486.87	495.28	530.61	531.98
	FWHM	1.25	1.23	1.32	2.47
	Area	1682.83	1117.89	532.79	0.00
	Lineshape	LF	LF	LF	LA
D5	BE [eV]	486.65	495.07	530.38	532.34
	FWHM	1.22	1.19	1.37	1.34
	Area	1049.50	700.70	355.85	14.11
	Lineshape	LF	LF	LF	LA

Table 8: Fit parameters of the 2000 eV 'deep' spectra in the HIGH_T experiment.

Step	Parameter	Sn 3d 5/2	Sn 3d 3/2	O lattice	O third
UHV before	BE [eV]	487.01	495.44	530.87	N/A
	FWHM	1.43	1.40	1.41	N/A
	Area	7367.83	5062.35	2295.80	0.00
	Lineshape	LF	LF	LF	LA
D1	BE [eV]	496.75	495.18	530.66	532.05
	FWHM	1.35	1.33	1.40	1.76
	Area	6310.89	4301.78	2022.33	20.92
	Lineshape	LF	LF	LF	LA
E1	BE [eV]	486.94	495.39	530.89	532.15
	FWHM	1.38	1.35	1.42	2.50
	Area	7344.72	5019.57	2362.35	18.19

	Lineshape	LF	LF	LF	LA
D2	BE [eV]	486.77	495.20	530.70	532.79
	FWHM	1.33	1.32	1.44	0.80
	Area	6332.46	4318.40	2137.47	7.04
	Lineshape	LF	LF	LF	LA
E2	BE [eV]	487.08	495.44	530.94	532.70
	FWHM	1.39	1.36	1.43	0.90
	Area	7349.93	4973.67	2131.00	16.71
	Lineshape	LF	LF	LF	LA
D3	BE [eV]	486.64	495.06	530.52	532.29
	FWHM	1.36	1.32	1.37	0.80
	Area	4973.67	4391.26	2218.94	6.80
	Lineshape	LF	LF	LF	LA
D3.5	BE [eV]	486.75	495.17	530.64	532.71
	FWHM	1.33	1.31	1.39	0.80
	Area	5943.98	4079.86	2109.74	17.61
	Lineshape	LF	LF	LF	LA
E3	BE [eV]	486.95	495.37	530.83	N/A
	FWHM	1.38	1.36	1.45	N/A
	Area	7492.91	5096.49	2524.30	0.00
	Lineshape	LF	LF	LF	LA
D4	BE [eV]	486.73	495.16	530.59	532.66
	FWHM	1.36	1.33	1.41	0.80
	Area	6615.94	4511.43	2263.40	4.90
	Lineshape	LF	LF	LF	LA
D4.5	BE [eV]	486.72	495.12	530.66	N/A
	FWHM	1.33	1.32	1.38	N/A
	Area	5618.68	3850.77	2263.40	0.00
	Lineshape	LF	LF	LF	LA
E4	BE [eV]	486.87	495.30	530.75	N/A
	FWHM	1.37	1.33	1.44	N/A
	Area	6878.80	4615.15	2351.02	0.00
	Lineshape	LF	LF	LF	LA
D5	BE [eV]	486.74	495.16	530.63	N/A
	FWHM	1.34	1.30	1.38	N/A
	Area	5636.31	6846.45	1962.17	0.00
	Lineshape	LF	LF	LF	LA

Disregarded resistance measurements:

Typically, the resistance of n-type semiconductors, such as SnO_2, increases with increasing oxygen pressure and decreases in UHV, provided that the temperature is high enough. While an increase in resistance was observed during D1, upon going to step E1, the sensor's resistance increased further, initially up to 8.5 kΩ and then decreased gradually to reach 8 kΩ. The reintroduction of O_2 caused the sensor's resistance to decrease to a value comparable to D1, around 5 kΩ. Similar behaviour was observed during the next two steps, where removal of O_2 caused an increase in resistance up to 10 kΩ during E2 and a decrease to 6 kΩ during D3. Such behaviour is in conflict with the current understanding of the effect of oxygen on the resistance of n-type semiconductors. While O_2

pressure-dependent n-p switching was observed before in some semiconducting metal oxides, this is typically explained in terms of the n-type semiconductor becoming sufficiently electron depleted due to the large density of surface acceptors that instead electron holes become the dominant charge carriers, resulting in a switch to p-type at high O_2 pressures.[9,10] However, the described behaviour is different to that observed here, where the p-type response (resistance decrease with increasing pO_2, E1 to D2 and E2 to D3) is observed at lower O_2 pressure (UHV to 1 mbar) and the n-type response is observed at a higher pressure (resistance increase from 1 mbar to 2 mbar, D3 to D3.5). To the best of our knowledge, such behaviour has not been reported in the literature and we are unable to provide an explanation at present. Therefore, the affected resistance measurements were disregarded and marked in grey on the macroscopic analysis plot (Figure S9, bottom panel).

Considerations on the origin of 'O third' in HIGH_T

The same O_2 gas, containing sub-ppm levels of H_2O and H_2, was used in both the LOW_T and HIGH_T experiments. Therefore, as discussed in the case of LOW_T, surface hydroxyls are unlikely to be responsible for the 'O third' emission in the HIGH_T spectra. Further evidence is provided for the O third assignment NOT being related to surface hydroxyls in the HIGH_T experiments because the O third peak disappears entirely at 350 °C; a temperature of 400 °C is necessary to start removing surface hydroxyls from SnO_2, and at least 500 °C for a complete dehydroxylation of the surface.[11,12] Indeed, in previous experiments we observed that the O 1s peak asymmetry attributable to surface hydroxyls persists during heating in UHV up to 350 °C, indicating that higher temperatures are necessary to remove the surface species. The next obvious candidate, oxygen contained in adventitious carbon contamination, must also be excluded based on the spectra presented in Figure S10. The contaminants, as deposited onto the surface during reduction, are composed mainly of C-H carbon, with some contribution from C-OH and virtually no C=O or O-C=O. As a result of O_2 exposure during D1, the carbon is oxidised, and the corresponding peaks increase in intensity at the expense of the C-H peak. After reaching their maximum abundance around steps D1/E1, the oxidised organic contaminants are gradually removed and disappear almost entirely by the end of the experiment. To show this quantitatively, relative to the highest estimate obtained (D1), the amount of organic oxygen on the surface in D2, D3, D4 and D5 decreases to 0.74, 0.47, 0.22 and finally 0.19. This trend is clearly different from the behaviour of the 'O third' peak, which alternates between two relatively constant values depending on O_2 exposure. Therefore, the peak cannot be ascribed to the oxygen atoms contained in adventitious carbon contamination, and it is concluded that oxygen adsorbates are the main contributor to the 'O third' emission.

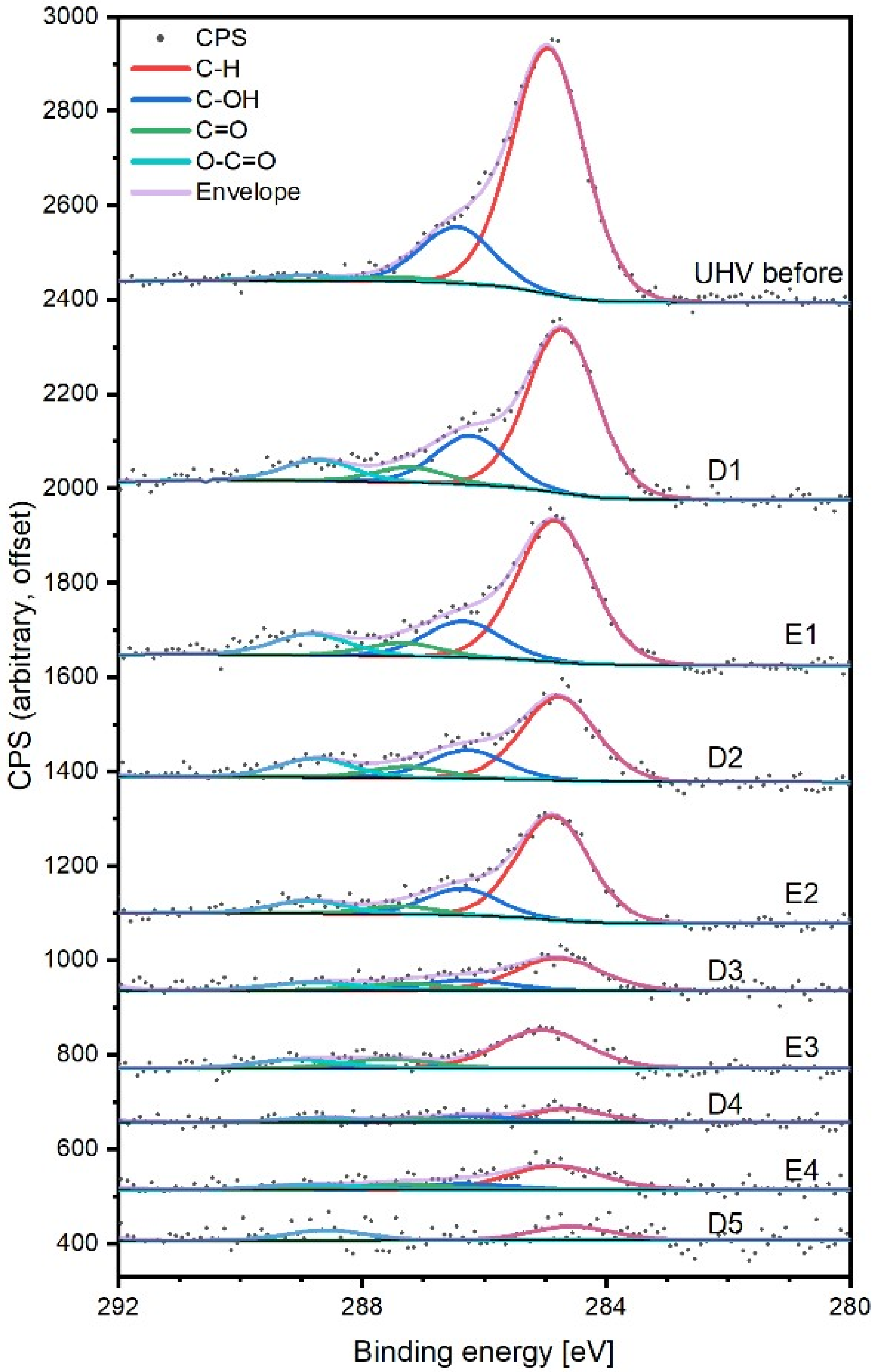

Figure S10: Comparison of the C 1s emission (collected with a 30 eV pass energy) during HIGH_T. After the initial increase, the amount of oxidised organic contamination on the surface decreases steadily throughout the experiment. CPS was normalised with respect to the corresponding Sn 3d peaks to ensure intensity comparability. Additionally, the spectra collected in 1 mbar O2 (D#) were corrected for signal attenuation by gas molecules – for details, see Experimental Section.

References

[1] G. Held, F. Venturini, D. C. Grinter, P. Ferrer, R. Arrigo, L. Deacon, W. Quevedo Garzon, K. Roy, A. Large, C. Stephens, A. Watts, P. Larkin, M. Hand, H. Wang, L. Pratt, J. J. Mudd, T. Richardson, S. Patel, M. Hillman, S. Scott, *J. Synchrotron Radiat.* **2020**, *27*, 1153–1166.

[2] A. Klein, T. Mayer, A. Thissen, W. Jaegermann, in *Methods Phys. Chem.*, Wiley-VCH Verlag GmbH & Co. KGaA, Weinheim, Germany, **2012**, pp. 477–512.

[3] S. Tanuma, C. J. Powell, D. R. Penn, *Surf. Interface Anal.* **1988**, *11*, 577–589.

[4] S. Kucharski, C. Blackman, *Chemosensors* **2021**, *9*, 270.

[5] W. M. Hlaing Oo, S. Tabatabaei, M. D. McCluskey, J. B. Varley, A. Janotti, C. G. Van de Walle, *Phys. Rev. B* **2010**, *82*, 193201.

[6] Z. Lu, D. Ma, L. Yang, X. Wang, G. Xu, Z. Yang, *Phys. Chem. Chem. Phys.* **2014**, *16*, 12488–12494.

[7] J.-M. Ducéré, A. Hemeryck, A. Estève, M. D. Rouhani, G. Landa, P. Ménini, C. Tropis, A. Maisonnat, P. Fau, B. Chaudret, *J. Comput. Chem.* **2012**, *33*, 247–258.

[8] M. Eslamian, A. Salehi, E. Nadimi, *Surf. Sci.* **2021**, *708*, 121817.

[9] A. Gurlo, N. Bârsan, A. Oprea, M. Sahm, T. Sahm, U. Weimar, *Appl. Phys. Lett.* **2004**, *85*, 2280–2282.

[10] D. E. Williams, P. T. Moseley, *J. Mater. Chem.* **1991**, *1*, 809.

[11] N. Yamazoe, J. Fuchigami, M. Kishikawa, T. Seiyama, *Surf. Sci.* **1979**, *86*, 335–344.

[12] E. Leblanc, L. Perier-Camby, G. Thomas, R. Gibert, M. Primet, P. Gelin, *Sensors Actuators, B Chem.* **2000**, *62*, 67–72.
